# Efficacy of combined tumor irradiation and K_Ca_3.1-targeting with TRAM-34 in a syngeneic glioma mouse model

**DOI:** 10.1038/s41598-023-47552-4

**Published:** 2023-11-23

**Authors:** Nicolai Stransky, Katrin Ganser, Leticia Quintanilla-Martinez, Irene Gonzalez-Menendez, Ulrike Naumann, Franziska Eckert, Pierre Koch, Stephan M. Huber, Peter Ruth

**Affiliations:** 1 Department of Radiation Oncology, University of Tübingen, Hoppe-Seyler-Str. 3, 72076 Tübingen, Germany; 2 Department of Pharmacology, Toxicology and Clinical Pharmacy, Institute of Pharmacy, University of Tübingen, 72076 Tübingen, Germany; 3 Institute of Pathology and Neuropathology, Comprehensive Cancer Center, Eberhard Karls University of Tübingen, 72076 Tübingen, Germany; 4 Cluster of Excellence iFIT (EXC 2180) “Image-Guided and Functionally Instructed Tumor Therapies”, Eberhard Karls University, Tübingen, Germany; 5 Molecular Neurooncology, Hertie Institute for Clinical Brain Research and Center Neurology, University of Tübingen, 72076 Tübingen, Germany; 6 Faculty of Medicine University, Gene and RNA Therapy Center (GRTC), Tübingen, Germany; 7 Department of Radiation Oncology, Comprehensive Cancer Center, Medical University Vienna, AKH, Wien, Austria; 8 Department of Pharmaceutical/Medicinal Chemistry II, Institute of Pharmacy, University of Regensburg, 93040 Regensburg, Germany

**Keywords:** CNS cancer, Radiotherapy, Cancer microenvironment

## Abstract

The intermediate-conductance calcium-activated potassium channel K_Ca_3.1 has been proposed to be a new potential target for glioblastoma treatment. This study analyzed the effect of combined irradiation and K_Ca_3.1-targeting with TRAM-34 in the syngeneic, immune-competent orthotopic SMA-560/VM/Dk glioma mouse model. Whereas neither irradiation nor TRAM-34 treatment alone meaningfully prolonged the survival of the animals, the combination significantly prolonged the survival of the mice. We found an irradiation-induced hyperinvasion of glioma cells into the brain, which was inhibited by concomitant TRAM-34 treatment. Interestingly, TRAM-34 did neither radiosensitize nor impair SMA-560’s intrinsic migratory capacities in vitro. Exploratory findings hint at increased TGF-β1 signaling after irradiation. On top, we found a marginal upregulation of *MMP9* mRNA, which was inhibited by TRAM-34. Last, infiltration of CD3^+^, CD8^+^ or FoxP3^+^ T cells was not impacted by either irradiation or K_Ca_3.1 targeting and we found no evidence of adverse events of the combined treatment. We conclude that concomitant irradiation and TRAM-34 treatment is efficacious in this preclinical glioma model.

## Introduction

Glioblastoma patients face a poor prognosis. Median survival times are in the range of 15–18 months in clinical trial settings^1^, with only around 7% of patients surviving longer than 5 years^2^. Several potential new therapeutics failed to prolong survival in recent randomized controlled trials: anti-VEGF antibody bevacizumab^3^, integrin inhibitor cilengitide^4^, EGFRvIII vaccination rindopepimut^5^ or anti-PD-1 antibody nivolumab^6,7^. Except for the implementation of Tumor Treating Fields electrotherapy^8^, the glioblastoma treatment protocol has not changed substantially in the last 15 years. It still comprises surgical resection, radiotherapy plus concomitant and adjuvant temozolomide chemotherapy^9^. Identifying new potential treatment targets may be an important step to overcome this standstill.

As such, one potential target may be the intermediate-conductance, calcium-activated potassium channel K_Ca_3.1 (also known as IK, IK_Ca_, or Gardos channel) encoded by the *KCNN4* gene. Promising results of the brain-penetrant^10,11^ K_Ca_3.1 channel inhibitor TRAM-34 in glioma cells, suggesting radiation-^12–14^ or temozolomide-sensitizing^15^ effects, were reported previously. Recent work of our group also elucidated radiation- and temozolomide-sensitizing effects (as well as direct tumoricidal effects) of TRAM-34^16^. However, these findings were only seen in certain glioma cell lines under specific cell culture conditions, questioning the general applicability of this potential treatment strategy. Beyond its proposed radio- and chemoresistance-conferring actions, K_Ca_3.1’s role in cell migration and invasion is well documented^17–19^, and K_Ca_3.1 targeting therapies were found to decrease glioma cell migration and invasion both in vitro and in vivo^10,20,21^. Last, new findings hint towards K_Ca_3.1’s important role in intertumoral communication networks, ultimately boosting tumor growth and potentially explaining TRAM-34’s glioma growth-inhibiting effects^22^.

Thorough assessments of K_Ca_3.1 targeting in glioma also need to study its off-tumor effects. While some reports found increased expression of K_Ca_3.1 in glioma cells compared to healthy tissues of the brain^19^, K_Ca_3.1 is also expressed in several normal cells, such as epithelia, endothelia or fibroblasts^23^, but also astrocytes or neurons^24,25^. Most importantly, it is functionally expressed in several immune cells and mediates important functions, such as T cell migration, activation and proliferation, or cytokine production and chemotaxis of macrophages^26,27^. Several studies tested TRAM-34 as an immunosuppressive, anti-inflammatory agent in autoimmune-like diseases, such as colitis^28^, asthma^29^ or allograft vasculopathy^30^. Additionally, recent findings in head and neck cancer patients showed that the immunosuppressing effects of immune checkpoints were (partly) driven by inhibition of K_Ca_3.1 channel activity^31^. Other reports indicate the important function of K_Ca_3.1 in CD8^+^ cytotoxic T cells’ infiltration into tumors^32^. All of the just-mentioned effects could potentially limit TRAM-34’s use as an anti-cancer therapeutic. However, two studies, testing TRAM-34 in immunocompetent mouse glioma models, found some evidence for potential immune-modulating (anti-tumor) effects^33,34^.

In the present study, we set out to test the effect of TRAM-34 in combination with fractionated irradiation on survival, glioma cell invasion and the tumor immune microenvironment in an immune-competent glioma model. We provide evidence of an irradiation-induced hyperinvasion of glioma cells, which was blunted by TRAM-34 and coincided with prolonged survival times. Furthermore, we did not detect differences of either treatment on the tumor immune microenvironment or blood counts of immune cells.

## Results

**Figure 1:**
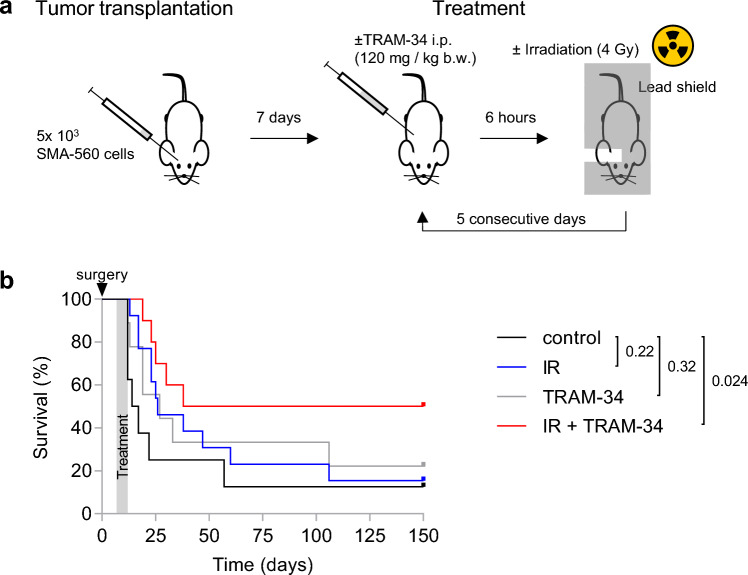
Combined irradiation + TRAM-34 therapy increases survival in the SMA-560 VM/Dk glioma model. (**a**), Schematic of tumor transplantation and subsequent treatment. (**b**), Kaplan–Meier estimator started on the day of tumor cell injection into the right striatum. Arrowhead depicts day of surgery; the treatment period is shaded in grey. Animals in the control group (N = 8) are shown in black, in the irradiation group (N = 13) in blue, in the TRAM-34 only (N = 10) group in grey and in the combined irradiation + TRAM-34 group (N = 9) in red. Numbers in (**b**) indicate *P* values as calculated by log-rank Mantel-Cox test.

K_Ca_3.1 targeting has been proposed to radiosensitize glioblastoma cells^12,13^ and to inhibit dissemination of glioblastoma cells in the brain^20^. Since K_Ca_3.1 is also highly expressed in immune cells^26,27^, this targeted therapy might interfere with the anti-glioblastoma immune response. We, therefore, studied the effect of K_Ca_3.1 targeting on the survival of tumor-transplanted mice, on the tumor dissemination in the brain, and on the immune-microenvironment in a syngeneic orthotopic glioma mouse model. To this end, we injected 5 × 10^3^ SMA-560 cells into the right striatum of VM/Dk mice (Fig. 1a). SMA-560 cells functionally express TRAM-34-sensitive K_Ca_3.1 channels in vitro (Supplemental Fig. S1, S2, S3), which is retained after tumor formation in VM/Dk mice (Supplemental Fig. S4). Gliomas were then treated by fractionated irradiation (5 × 0 or 5 × 4 Gy) concomitant to the systemic application of TRAM-34 (0 or 120 mg/kg b.w. in miglyol intraperitoneally [i.p.]; Fig. 1a) on five consecutive days.

Almost all animals, irrespective of treatment groups, lost weight during treatment (most notably starting on day 3 of the treatment). Weight loss may be due to daily intraperitoneal injections, daily isoflurane anesthesia or daily transport of the animals to the linear accelerator, all of which could ultimately elicit a stress response in the animals (see Supplemental Fig. S5). On the other hand, we detected no differences in weight loss between the treatment groups, which suggests no severe toxicity of the treatment.

In the control group, the tumor-transplanted mice exhibited a median survival of 16 days, which is comparable to other reports (16–27.5 days^35–37^). Neither irradiation only, nor TRAM-34 only did meaningfully affect the survival of the animals (median survival times: 26 and 27 days, respectively). Combined IR + TRAM-34, however, significantly prolonged the survival of the mice (median survival 38 days; Fig. 1b).

**Figure 2:**
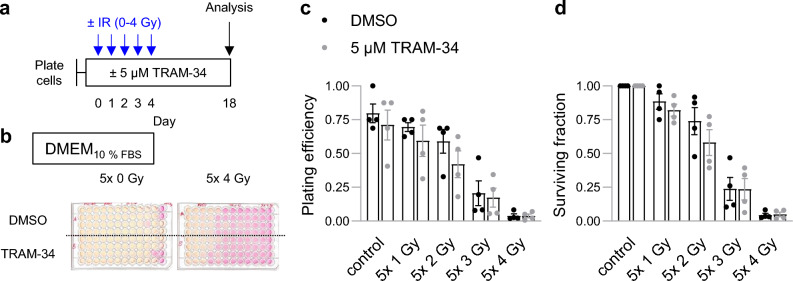
TRAM-34 does not affect clonogenic survival after fractionated irradiation in SMA-560 cells in vitro. The results shown are for SMA-560 cells grown in 10% FBS-containing DMEM. **a**, Scheme depicting the time course of the experiment. (**b**), Representative image of limited dilution assay on day 18 of cells treated with DMSO (upper wells) or 5 µM TRAM-34 (lower wells) and irradiation (left: 5 × 0 Gy; right: 5 × 4 Gy). (**c**), Plating efficiency and, (**d**), surviving fraction of cells after fractionated irradiation (5 × 0, 1, 2, 3 or 4 Gy) and TRAM-34 treatment (0 or 5 µM). (**c**, **d**), Individual values of 4 independent experiments and mean values ± standard error of the mean (sem) are depicted.

To identify potential mechanisms underlying the prolonged survival of the combined irradiation + TRAM-34 treated mice, we first determined the effect of K_Ca_3.1 channel blockade on the radiosensitivity of SMA-560 cells in vitro. Irradiation (2 Gy) increased TRAM-34-sensitive channel activity of K_Ca_3.1 in SMA-560 cells (see Supplemental Figures S1 and S2), which conferred radioresistance in other glioblastoma models^12,38^. A recent in vitro study of our group, however, concluded that TRAM-34 neither impairs clonogenic survival nor radioresistance in SMA-560 cells when treated with single-dose irradiation^16^. To exclude differential effects of TRAM-34 after fractionated irradiation protocols, we analyzed clonogenic survival of SMA-560 cells treated with fractionated irradiation (ranging from 5 × 0 to 5 × 4 Gy) and TRAM-34 (0 or 5 µM). Notably, fractionated irradiation with 5 × 4 Gy led to strong decreases in survival fraction. On the other hand, we did not detect meaningful effects of additional TRAM-34 treatment in standard (DMEM; Fig. 2) or glioblastoma stem-cell-enriching NSC medium (see Supplemental Fig. S6). This led us to assume that effects other than radiosensitization are responsible for the prolonged survival after combined IR + TRAM-34 treatment.

**Figure 3:**
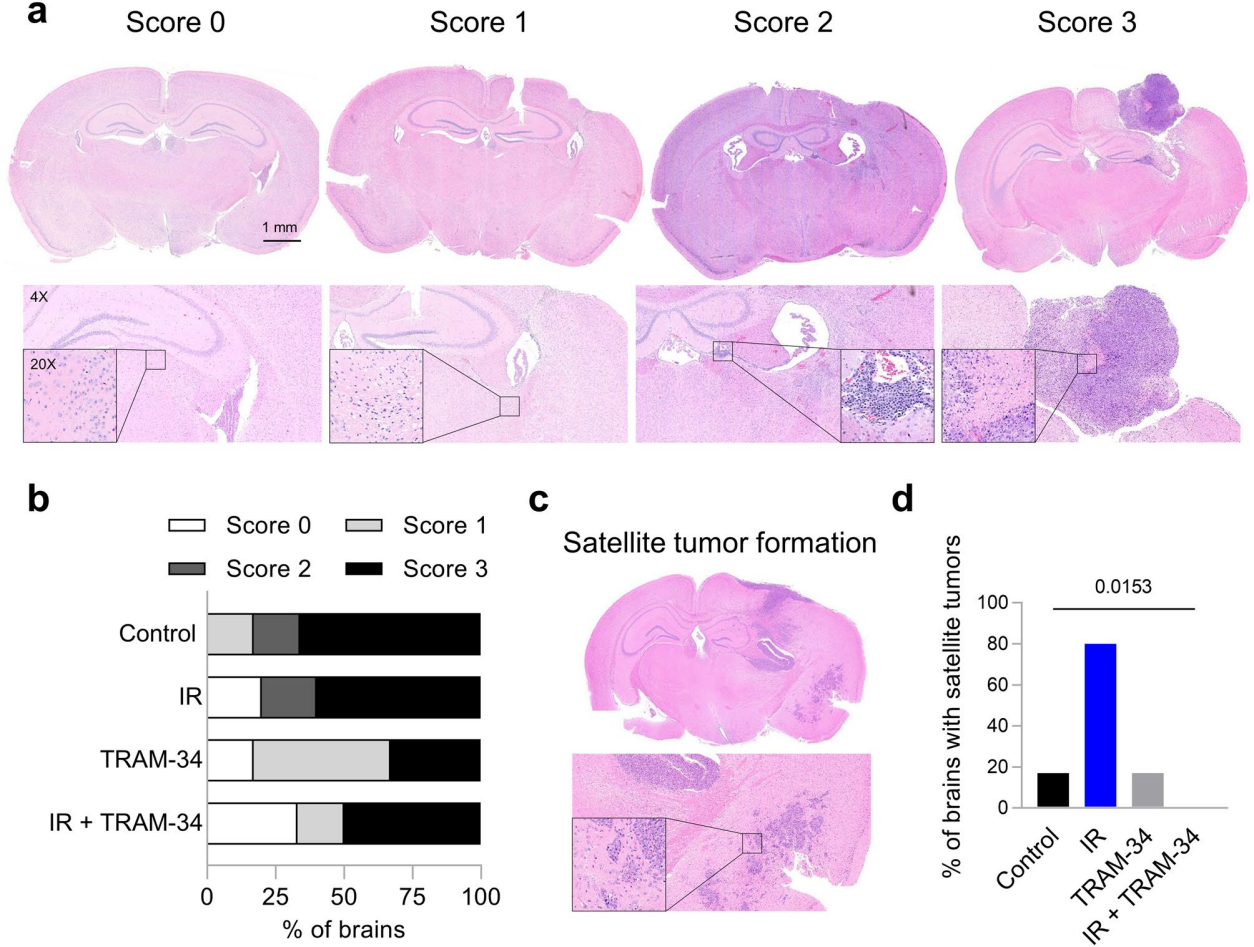
Irradiation-induced micro-satellite formation of SMA-560 gliomas is inhibited by TRAM-34. (**a**), Representative images of each score to subclassify tumor growth morphology in lowest (1x, top row), 4× (bottom row) or 20 × magnification (bar indicates 1 mm). Brains were classified as showing no difference to the contralateral hemisphere (Score 0), gliosis (Score 1), small diffuse tumors (Score 2) or medium-large solid tumor formation with necrosis (Score 3). (**b**), Quantification of each category among the four treatment arms (control: N = 6 animals; IR only: N = 5 animals; TRAM-34 only: N = 6 animals; IR + TRAM-34: N = 6 animals). (**c**, **d**), Satellite tumor formation was mostly found in animals from the irradiation only group, whereas no (0/6) animal from the combined IR + TRAM-34 treatment group exhibited this glioblastoma growth morphology. Number indicates *P* value as calculated by two-tailed Fisher’s Exact test (**d**).

Mesenchymal subpopulations of primary glioblastoma cells exhibit a highly invasive behavior in vitro and show an upregulation of K_Ca_3.1^39^. Moreover, K_Ca_3.1 targeting delayed glioblastoma brain invasion in an orthotopic *xeno*graft mouse model^10^ and after previous radiation therapy^21^. Hence, we analyzed the tumor growth morphology after up to 14 days after the end of treatment (Fig. 3). As soon as the first animal became symptomatic (Score ≥ 5, see methods), all animals of a simultaneously tumor-challenged batch of mice were sacrificed and brains were extracted. Some animals (especially in the TRAM-34 or combined IR + TRAM-34 treatment group) showed no signs of residual tumor (histological Score 0) or only gliosis (Score 1), whereas most animals presented with small (Score 2) to medium-large-sized tumors with necrosis (Score 3). Several animals developed satellite tumors, i.e. tumor cell clusters that were distant from the main tumor (Fig. 3c). Satellite tumors were frequently found in the IR only group (4/5 animals). In comparison, only 1/6 animals in the control and TRAM-34 only groups, and none (0/6 animals) in the combined IR + TRAM-34 treatment arm developed satellite tumors (Fig. 3d). Combined, these results point towards an irradiation-induced hyperinvasion of SMA-560 cells, which was completely blocked by TRAM-34 treatment in vivo.

**Figure 4:**
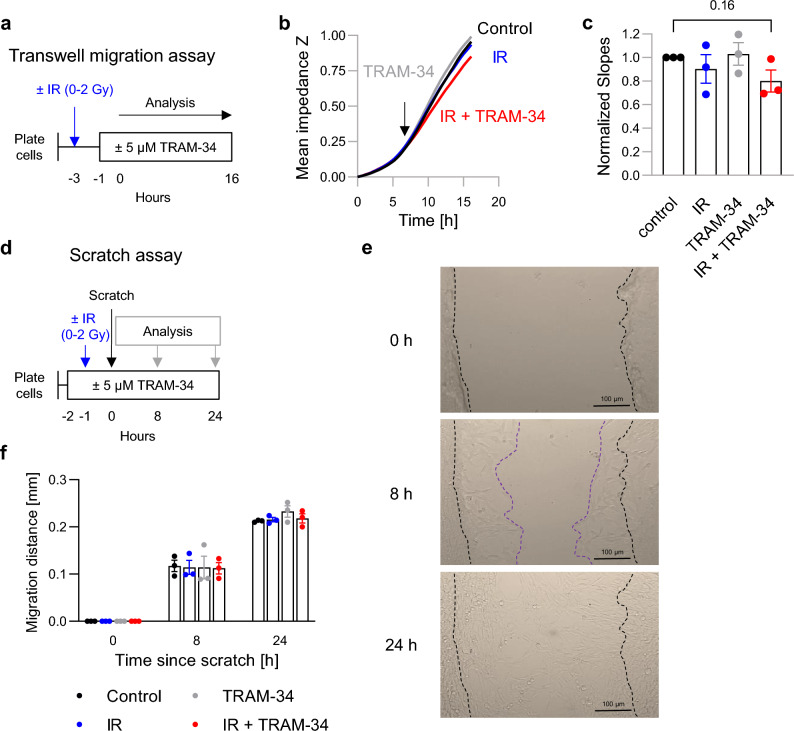
Combined single-dose irradiation (0 or 2 Gy) + TRAM-34 (0 or 5 µM) treatment does not affect SMA-560 cell migration in vitro. (**a**–**c**), Transwell migration assay with SMA-560 cells cultured in DMEM medium. (**a**), Scheme depicting time course of transwell migration assay. **b**, Mean impedance (as a measure of number of trans-migrated cells) over time averaged from three independent experiments. Arrow indicates the time point after which a linear course was assumed and the slope of the time-dependent impedance-increase calculated. **c**, Normalized slopes were not significantly different between treatment arms. Depicted are individual and mean values ± sem. (**d–f**), Scratch Assay with SMA-560 cells cultured in DMEM medium. (**d**), Scheme depicting time course of scratch assay. **e**, Representative images of scratches 0, 8 and 24 h after the scratch. The dashed black line shows the original distance of scratch, whereas the dashed purple line represents the scratch distance after 8 h. (**f**), Quantification of migration distance in all four treatment arms. Depicted are individual values from 3 independent experiments and means ± sem. Number indicates *P* value as calculated by two-tailed one-sample t-test against 1.0 (**c**).

To identify potential underlying processes, the effects of irradiation (1 × 0 Gy or 1 × 2 Gy) and TRAM-34 (0 or 5 µM TRAM-34) on migration velocity of SMA-560 cells was tested in vitro by transwell migration and wound healing assays (Fig. 4). Low irradiation doses were chosen due to findings of other authors, indicating increased invasion velocity of SMA-560 cells after low dose irradiation (2 Gy) compared to no increases after high irradiation doses (8 Gy)^35^. Overall, neither of the two assays disclosed an irradiation-induced hypermigration in vitro. Moreover, we did not observe any difference in transwell migration (Fig. 4a–c) and wound healing (Fig. 4d–f) between the four experimental arms. Only the combined treatment (1 × 2 Gy + 5 µM TRAM-34) showed a slight tendency (*P* = 0.16) towards lower transwell migration velocity (Fig. 4b,c). Together, these data suggest that satellite tumor formation after irradiation (Fig. 3c) is not due to an increase in intrinsic cell motility of SMA-560 cells.

**Figure 5:**
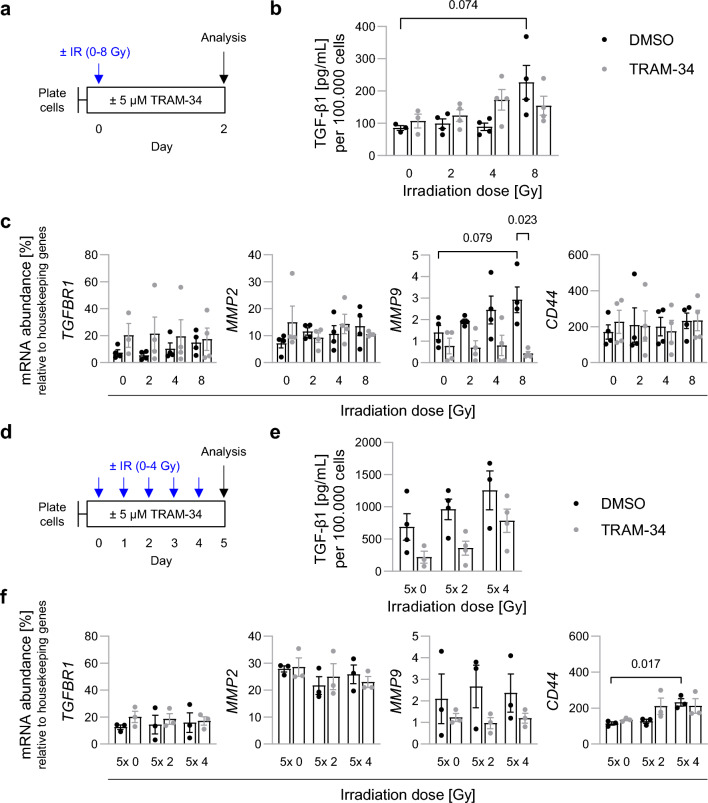
Irradiation increases TGF-β1 signaling and TRAM-34-sensitive *MMP9* mRNA abundance. (**a**–**c**), Effects of single-dose irradiation in SMA-560 cells cultured in DMEM medium. (**a**), Scheme depicting time course. (**b**), TGF-β1 secretion per 100.000 cells trends towards irradiation-induced increases at high irradiation doses as measured by ELISA with mixed effects of concomitant TRAM-34 treatment. **c**, mRNA abundance of *TGFBR1*, *MMP2*, *MMP9* and *CD44* as measured with RT qPCR relative to housekeeper genes. *MMP9* abundance is slightly increased after irradiation, whereas TRAM-34 consistently decreases *MMP9* expression. (**d**–**f**), Effects of fractionated irradiation in SMA-560 cells cultured in DMEM medium. (**d**), Scheme depicting time course. (**e**), Fractionated irradiation marginally increases TGF-β1 secretion per 100.000 cells (non-significant; n.s.), which is blunted by TRAM-34 (n.s.). (**f**), mRNA abundance of *TGFBR1*, *MMP2*, *MMP9* and *CD44* as measured with RT qPCR relative to housekeeper genes. Depicted are individual values from 3–4 independent experiments and mean values ± sem. Numbers indicate *P* values as calculated by Welch-corrected two-tailed t-tests (**b**, **c, f**).

Next, we analyzed further factors that might promote TRAM-34-sensitive radiogenic formation of SMA-560 microsatellites in vivo. Among those, we focused on auto-/paracrine TGF-β1 signaling, the interaction between CD44 and extracellular hyaluronic acid, and matrix metalloproteinases (MMP)-mediated remodeling of the tumor microenvironment^40^. Irradiation has previously been demonstrated to stimulate TGF-β signaling in orthotopic SMA-560 gliomas^35^, whereas pharmacological TGF-β targeting was shown to block invasion of SMA-560 cells in Boyden chamber experiments in vitro as well as microsatellite formation in vivo^36^. In our experiments, single-dose irradiation (Fig. 5a) induced a non-significant (*P* = 0.074) increase in TGF- β1 protein release at the highest irradiation dose in DMEM-grown SMA-560 cells. This trend was not observed when TRAM-34 was co-applied (Fig. 5b). In stem-cell-enriched SMA-560 cultures (NSC medium), single-dose (8 Gy; Supplemental Fig. S7a) or fractionated irradiation (5 × 4 Gy; Supplemental Fig. S7b) induced a profound (p < 0.005) but TRAM-34-insensitive increase in TGF-β1 protein release into the medium. Additionally, 8 Gy single-dose irradiation induced a non-significant (*P* = 0.079) rise in *MMP-9* mRNA abundance, which was blunted by TRAM-34 co-application. In contrast, no irradiation- or TRAM-34-modulated change in *TGF-β receptor-1* (*TGFBR1*), *MMP-2*, or *CD44* mRNA abundance was apparent after single-dose irradiation (Fig. 5c). Similar trends were observed when DMEM-grown SMA-560 cells were subjected to fractionated irradiation, except for an additional TRAM-34-insensitive rise in *CD44* mRNA only after 5 × 4 Gy irradiation (*P* = 0.017; Fig. 5d–f). Combined, these explorative experiments might hint at a radiogenic upregulation of auto-/paracrine TGF-β1 signaling and a TRAM-34-sensitive MMP-9-mediated remodeling of the tumor microenvironment.

**Figure 6:**
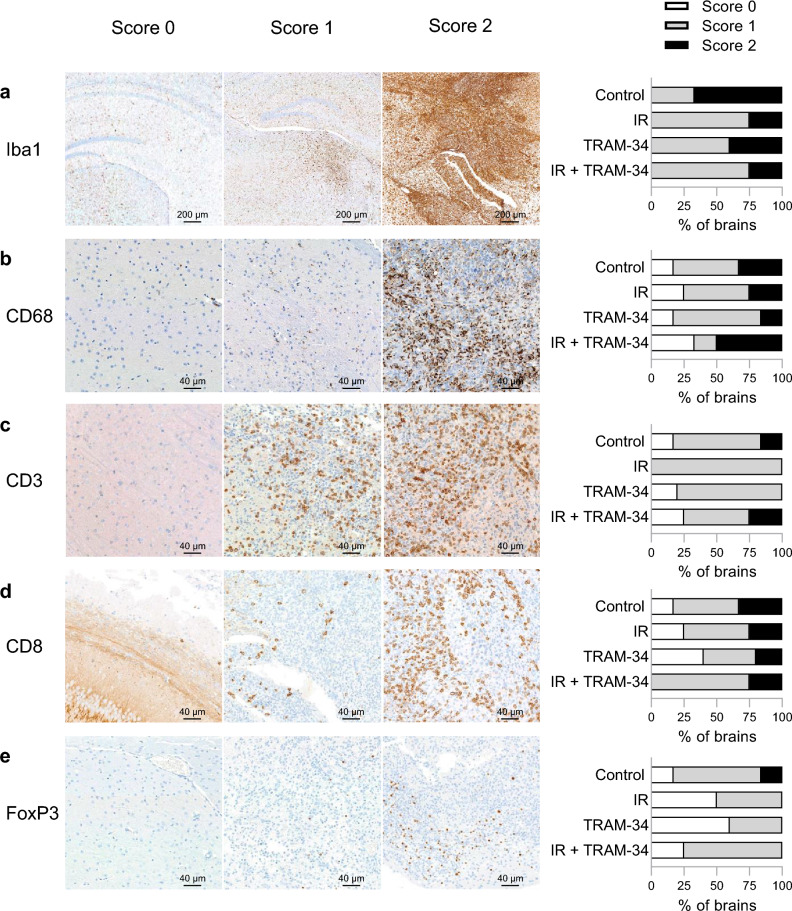
Immunohistochemical stains show no difference between treatment arms in the tumor-microenvironment. A scoring system to compare effects of the treatments was developed for, (**a**), Iba1^+^ reactive macrophages, (**b**), CD68^+^ reactive macrophages, (**c**), CD3^+^ T cells, (**d**), CD8^+^ cytotoxic T cells and **e**, FoxP3^+^ regulatory T cells. No animal with an H&E score of > 0 displayed an Iba1 Score of 0. Additionally depicted is the share of animals per treatment group displaying each score (right side; control: N = 6 animals; irradiation only: N = 4 animals; TRAM-34 only: N = 5 animals; IR + TRAM-34: N = 4 animals).

Since TGF-β has also been reported to exert several immuno-suppressive functions^41^ and K_Ca_3.1 has been proposed to exert pivotal functions in the anti-cancer immune response^31,32^ or T cell migration^27^ on its own, we next analyzed the effects of fractionated irradiation and K_Ca_3.1 targeting on the immune microenvironment of the SMA-560 glioma mouse model. As expected, an increase in the number of Iba1^+^ reactive microglia cells in or around the tumor (Score 1; ‘gliosis’) was detected in the majority of animals as compared to the healthy contralateral hemisphere. We found a moderate to prominent Iba1^+^ macrophage reaction in larger solid tumors (Score 2, Fig, 6a), including macrophage pseudopalisading in several animals (Supplemental Fig. S8). Additionally, we used another macrophage activation marker, CD68. In general, we found less CD68^+^ than Iba1^+^ macrophages with little differences among the treatment groups (Fig. 6b). The amount of Iba1^+^ and CD68^+^ cells correlated with each other and the tumor size, i.e., bigger tumors contained more Iba1^+^ and more CD68^+^ macrophages. Furthermore, most animals presented with a mild-moderate amount of CD3^+^ T cells (Score 1), while 2 animals displayed a prominent presence of CD3^+^ T cells (Score 2; Fig. 6c, right), mostly at the tumor periphery (Supplemental Fig. S8). No differences were detected regarding the CD8 (Fig. 6d) and FoxP3 positive T cells (Fig. 6e) among the treatment groups.

Combined, these data indicate that neither fractionated radiation nor K_Ca_3.1 targeting with TRAM-34 nor their combination, induced pronounced changes in the abundance of Iba1^+^ or CD68^+^ reactive microglia and CD3^+^, CD8^+^ cytotoxic or FoxP3^+^ regulatory T cells in the SMA-560 VM/Dk glioma model.

**Figure 7:**
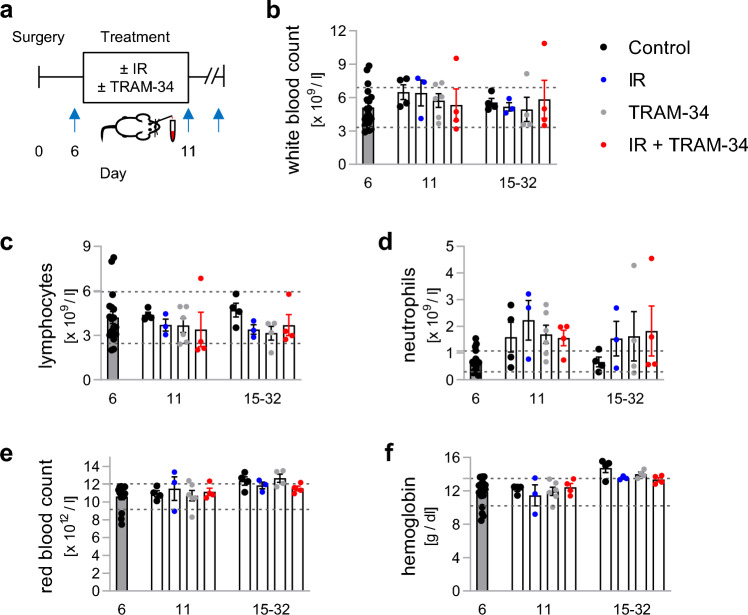
Fractionated irradiation and/or TRAM-34 have little effect on blood counts in the SMA-560 VM/Dk glioma model. (**a**), Scheme depicting time course of sample collection as indicated by blue arrows. (**b**–**f**), Depicted are individual values from 3 to 6 animals and mean values ± sem. Additionally, a ‘normal range’ of values (defined as the range of mean value ± standard deviation of all animals before the start of treatment) are outlined by dashed lines representing the upper and lower limit.

To analyze treatment-dependent systemic changes of the immune system, we finally determined the counts of total white blood cells (WBC), lymphocytes and neutrophils, shortly before (day 6), as well as directly (day 11) and 0.5–3 weeks (days 15–32) after the end of treatment (Fig. 7a). In addition, blood hemoglobin concentration and red cell count were used as markers of treatment-associated adverse events, as K_Ca_3.1 has previously been shown to be important for volume regulation in erythrocytes^42,43^. Blood counts of animals were well balanced at the time of randomization (see Supplemental Fig. S9) and were used to calculate ‘normal blood count ranges’ for the VM/Dk mice (as defined by mean values ± 1 standard deviation of all mice before treatment; indicated in Fig. 7 by dotted grey lines). According to this ‘normal range‘, white blood count (Fig. 7b) and lymphocytes (Fig. 7c) remained unchanged after the treatments, at most displaying slight reductions of WBC after combined IR + TRAM-34 or of lymphocytes after irradiation only, TRAM-34 only or combined IR + TRAM-34 treatment (all n.s., ordinary one-way ANOVA followed by Tukey’s multiple comparisons test). We observed an increase in neutrophil counts in each treatment group on day 11, whereas neutrophil counts at later time points varied considerably between the animals (Fig. 7d). Both red blood cell count (Fig. 7e) and hemoglobin concentration (Fig. 7f) remained unchanged at day 11 and appeared to slightly increase at later time points irrespective of treatment group. Overall, except for increases in neutrophil counts, arguably due to increased stress during the treatment, we found little to no effect of our treatment regimens on blood counts.

## Discussion

Our study provides evidence that fractionated tumor irradiation combined with systemic application of the K_Ca_3.1 blocker TRAM-34 prolongs the survival of VM/Dk mice with orthotopically transplanted syngeneic SMA-560 cells. In contrast, fractionated irradiation alone (5 × 4 Gy, corresponding to an equivalent dose in 2 Gy fractions [EQD2] of 23.3 Gy assuming an alpha/beta ratio of 10 Gy) did not meaningfully affect the survival of the animals (see Fig. 1b). These in vivo results are in line with other reports after single-dose irradiation with 12 Gy (EQD2 = 22 Gy), which also reported no meaningfully prolonged survival in this glioma model^35,44^. This contrasts findings of our in vitro experiments, showing 80–90% reductions in clonogenic survival of SMA-560 cells after 5 × 4 Gy irradiation treatment (see Fig. 2C, S4). Explanations for the discrepant in vivo and in vitro results may comprise hypoxic tumor cores in vivo, a well-known contributor to increased radioresistance^45,46^, or reciprocal interactions of SMA-560 glioma cells with the tumor microenvironment and stroma cells^47,48^. This may also be supported by increased radioresistance of stem-cell-enriched SMA-560 cells (NSC medium) compared to normal DMEM culture conditions^16^.

While TRAM-34 applied concomitantly to fractionated irradiation resulted in prolonged mouse survival (see Fig. 1b), the K_Ca_3.1 blocker did not radiosensitize SMA-560 cells in vitro in the present (see Fig. 2c, S6) or our recent study^16^. Radiosensitizing effects of TRAM-34 have been reported previously in different in vitro and ectopic mouse glioma models^12,14^. Hence, one might speculate that SMA-560 cells acquire a TRAM-34-sensitive radioresistant phenotype in an orthotopic microenvironment in vivo. Intriguingly and similarly to our results, targeting HGF/MET in combination with tumor irradiation (but not tumor irradiation alone) prolonged survival in the SMA-560 glioma model but did not affect radiosensitivity in vitro^35^. From our data, we cannot distinguish whether the blockade of mitochondrial^49^ or of plasmalemmal K_Ca_3.1 channels, or of both, contributed to these findings.

Our histological analysis provides evidence of a hyperinvasion of SMA-560 cells in the brain after irradiation (see Fig. 3c,d). This has been reported previously in vitro and in vivo in other glioma models^14,21,50,51^. As the occurrence of multifocal glioblastoma is associated with a worse prognosis^52^, this might also explain the lacking efficacy of radiation treatment alone in this glioma model. While irradiation will most likely reduce tumor growth in the irradiation field, this beneficial effect on mouse survival appears to be counteracted by a co-occurring hyperinvasion of cancer cells. Overall, a balance of both effects might result in similar survival times as compared to animals in the control arm.

Importantly, the concomitant application of TRAM-34 abolished the radiogenic hyperinvasion in the present study (see Fig. 3c,d). This might suggest that radiation treatment only resulted in meaningful survival benefits when radiogenic tumor spreading was inhibited by TRAM-34. K_Ca_3.1 has been demonstrated in various in vitro and in vivo models to be required for migration and brain invasion of glioblastoma cells^10,17,19–21^. Consistently, K_Ca_3.1 (*KCNN4*) mRNA abundance of primary glioblastoma stem cells correlated with mesenchymal marker expression and in vitro matrix invasion capability in our previous work^13,39^. In contrast, we found no irradiation-induced or TRAM-34-sensitive transfilter migration or wound healing of DMEM-grown SMA-560 cells in vitro (see Fig. 4). Hence, one might speculate that K_Ca_3.1 regulates 3D invasion (secretion of matrix metalloproteases, etc., see next paragraph) rather than migration mechanics in SMA-560 cells.

Auto-/paracrine TGF-β signaling might be a potential pathway involved in this process (albeit a significant radiogenic stimulation of TGF-β1 secretion was only found in stem-cell-enriched NSC cultures in vitro and was not TRAM-34 sensitive, see Fig. 5b–e, S7). Previous work has demonstrated that radiation indeed stimulates TGF-β signaling in the orthotopic SMA-560 glioma model via hepatocyte growth factor (HGF) and its receptor MET^35^. Matrix metalloproteinases (MMPs) are TGF-β downstream targets and are reportedly involved in glioblastoma brain invasion^53,54^. In the present study, a single dose (8 Gy) of irradiation led to a doubling of *MMP9* (*P* = 0.079) but not of *MMP2* mRNA abundance in DMEM-grown SMA-560 cells (see Fig. 5c). Importantly, TRAM-34 co-treatment decreased *MMP9* mRNA abundance after either single dose or fractionated irradiation treatment (Fig. 5c–f). This might hint at an activation of K_Ca_3.1 by irradiation, ultimately leading to TGF-β release, *MMP9* expression and increased cell invasion.

As mentioned above, immune cell function, e.g., migration or proliferation of T cells, may critically depend on K_Ca_3.1 channel activity^26,27^. In the present study, we detected no detrimental effects on immune cell infiltration or blood counts of TRAM-34 treatment + /- irradiation (see Figs. 6 and 7). Reassuringly, the invasion of cytotoxic CD8^+^ T cells was not blunted by irradiation and/or TRAM-34 treatment. On top, we detected no increase in immunosuppressive FoxP3^+^ regulatory T cells, in contrast to other authors’ conclusion that regulatory T cells may be more radioresistant and hence may accumulate after irradiation^55^. It appears tempting to speculate whether the prolongation of survival may partly be also due to TRAM-34’s immunomodulating effects in glioma as described elsewhere^33,34^. In this line, other authors concluded that not intrinsic radiosensitivity of cancer cells, but rather factors of the host immune system may correlate with the efficacy of radiotherapy^56^. In particular, the authors identified CD8^+^ T cells as essential contributors for successful radiotherapy in a murine breast cancer model, a conclusion similar to other work^57^. Our immunohistochemical analyses did by and large not identify differences in intratumoral CD8^+^ T cells between the treatment arms (see Fig. 6d), even though there exist multiple studies associating higher CD8^+^ counts with prolonged survival of glioblastoma patients^58–60^. New studies may try to elucidate immune cell subpopulations predictive of efficacious irradiation treatment in primary brain tumors.

Last, we did not detect safety signals when combining TRAM-34 with radiation treatment (see Fig. 7 and S5). This builds on previous evidence on the tolerability of K_Ca_3.1 targeting: both *KCNN4*^−/−^ animal models (except impaired volume control of erythrocytes and lymphocytes^42,43^), as well as clinical data on the structurally-related K_Ca_3.1 blocker senicapoc in patients with sickle cell disease did not detect serious adverse effects^61^.

There are several limitations to our work. We could not delineate the precise molecular mechanism driving the synergism of radiation + TRAM-34 treatment. Our results point towards irradiation-induced TGF-beta secretion, followed by hyperinvasion of tumor cells into the brain parenchyma, which might be inhibited by TRAM-34. However, one may debate whether the anti-invasive action of drugs is generally likely to translate into clinical efficacy. Most glioblastoma patients—even after complete macroscopic tumor resections—will ultimately relapse^62^. This finding hints at an early spreading of tumor cells, likely before the initial diagnosis has been made. Evidence of a whole brain invasion of glioma cells exists for *IDH*-mutated gliomas^63^. Similar explanations may apply to the failure of metastatic pathway targeting therapeutics (such as MMP inhibitors) in nearly all large randomized controlled trials in solid tumors so far^64,65^. Whereas treatment in preclinical tumor models may be started at a very early stage, this does not apply to cancer patients with visible primary tumors, arguably months or years after the metastatic (or invasive) process has started^66^. On a more general note, we only used one syngeneic glioma model limiting the generalizability of the results. Further experiments in other syngeneic glioma models, such as GL-261/C57BL/6 or CT-2A/C57BL/6^67^, are needed. Along those lines, acute inflammatory processes elicited by transplanting syngeneic glioma cells in the brains of immunocompetent mice differ substantially from longer-lasting immune editing processes occurring in glioblastoma patients^68^. Here, genetically induced glioma animal models might bring further insights^69–71^.

To conclude, K_Ca_3.1 targeting with TRAM-34 seems to inhibit invasion of irradiated SMA-560 glioma cells in vivo. Most importantly, these effects are relevant for the survival of the glioma mice and are not associated with apparent immune cell alterations. Due to lacking TRAM-34 effects in our in vitro experiments, it is tempting to speculate that the TRAM-34 sensitive phenotype is only acquired in the orthotopic syngeneic brain environment.

## Material and methods

All sections henceforth are written in accordance with MeRIT (Method Reporting with Initials for Transparency)^72^.

*Cell culture* Murine glioma cells SMA-560 were originally derived from a spontaneous murine astrocytoma in VM/Dk mice^73,74^. The cells were cultivated in DMEM medium (ThermoFisher, #41965-039) supplemented with 10% fetal bovine serum (FBS) in a 10% CO_2_ atmosphere if not otherwise specified. In experiments assessing the migratory properties of cells, FBS levels were lowered to 0.1–1% to reduce proliferative capacity while ensuring the viability of cells. To enrich SMA-560 stem cells (see Supplementary Figures S6, S7), SMA-560 cells were grown as spheroids in FBS-free human NeuroCult NS-A Proliferation Medium (including 10 ng/mL rhFGF, 20 ng/mL rhEGF, 2 µg/mL Heparin; STEMCELL Technologies, #05751, #78003, #78006.2, #07980) at 37 °C and 5% CO_2_.

Adherent cells were detached (DMEM medium) or cells separated from spheroids (NSC medium) with Trypsin–EDTA 0.05% (ThermoFisher, #25300054) and manually counted with Hemocytometer Chips (Neubauer improved).

*Drug treatment* TRAM-34 was synthesized in house by P.K. using the synthetic strategy reported by Wulff et al.^75^ The chemical purity and identity of TRAM-34 was confirmed by high-performance liquid chromatography (HPLC) and ^1^H-NMR (HPLC and NMR spectra can be found in the Supplementary Appendix). For in vitro experiments, TRAM-34 was dissolved in DMSO at concentrations of 1 or 10 mmol/L and used at final concentrations of 200 nM or 1 µM (patch clamp experiments) or 5 µM (all other experiments) with same volumes of DMSO serving as vehicle control. TRAM-34 concentrations used for in vitro experiments are in the same range as previously found in brain tissue of up to 1.3 µM in mice^10^ or up to 2.5 µM in rats^11^. 1-EBIO (1-ethylbenzimidazolinone, Sigma-Aldrich) was prepared from 200 mM stock solution in DMSO and used at final concentrations of 200 µmol/L for patch clamp experiments. For in vivo experiments, TRAM-34 was dissolved in miglyol-812 (Caesar & Loretz, PZN1115805) at a concentration of 12 mg/mL (34,8 mmol/L) before sterile filtration (Millex – FG 0.20 µm, SLFG025LS, Merck) resulting in injection volumes of 10 µL per gram of bodyweight. Accordingly, equal volumes of miglyol-812 were used as vehicle control. Except for patch clamp experiments, all experiments were conducted in a blinded fashion until data visualization or statistical analysis. Sketches depicting exact treatment times can be found in respective figures or in the following paragraphs.

*Irradiation.* Photon irradiation was performed by NS or KG with a 6 MV linear accelerator (LINAC SL25 Philips) at room temperature. The applied dose rate for in vitro experiments was 5 Gy/min. Equivalent dose in 2 Gy fractions (EQD2) was calculated with an online tool http://www.eqd2.de/ assuming an alpha/beta ratio of 10 Gy for SMA-560 cells.

*Limited dilution assays.* To assess the effect of fractionated irradiation in combination with 5 µM TRAM-34 on plating efficiency and survival fractions, limited dilution assays were performed by NS. Cells were plated in 96 well plates in serial dilutions (2048-1 cell per well). 4 h after plating the cells, TRAM-34 or vehicle (DMSO) was added. Irradiation treatment (0, 1, 2, 3 or 4 Gy) was started 1 h after addition of the drug, and repeated for another 4 consecutive days. 14 days after the last day of irradiation, numbers needed to retain culture (defined as cell colonies or spheroids consisting of at least 50 cells) were determined. Results of 4 wells per treatment group were averaged for each independent experiment. Plating efficiency and surviving fraction were calculated as follows:

$$Plating\,\, efficiency= \frac{1}{Number\,\, of\,\, cells\,\, needed\,\, to\,\, retain\,\, culture}$$

$$Survival\,\,fraction= \frac{Plating\,\,efficiency\,\,(treatment\, group \,X \,at\, Y \,Gy)}{Plating\,\,efficiency\,\,(treatment\,\,group\,\,X\,\,at\,\,0\,\,Gy)}$$

Of note, determining numbers needed to retain culture at other time points (e.g. 7 days, 21 or 28 days after the last day of irradiation) led to negligible differences in plating efficiency or survival fraction results (data not shown).

*Transwell migration.* The transwell migration of SMA-560 cells was measured using the Real-Time Cell Analyzer DP (xCELLigence, Roche, Mannheim, Germany) and performed by NS. After irradiation of cells (0 or 2 Gy), cells were detached and 40.000 cells in DMEM/1% FBS, further containing 5 µM TRAM-34 or vehicle (DMSO) alone, were pipetted into filter inserts forming the upper chambers of a CIM-Plate-16. The lower chambers were filled with DMEM/10% FBS to produce a chemoattractant transfilter gradient. Upon plugging the filter inserts into the lower chambers, the electrical impedance between gold electrodes at the outer face of the filter membrane and the bottom of the lower chamber was continuously recorded. Transfilter migration and subsequent cell settling on the gold electrodes of the filter membrane results in an increase in this impedance. As a measure of migration velocity, the slope of the time-dependent impedance increase was analyzed as soon as this slope became linear. Due to high inter-experimental scattering, recorded slopes had to be normalized to that of the respective control arm (vehicle, 0 Gy) to become comparable between independent experiments.

*Scratch assay (wound healing assay)* The scratch assay was performed by NS as previously described^76^. Briefly, 75.000 SMA-560 cells were plated into 96 well plates and let adhere overnight. To minimize proliferation of cells, 0.1% FBS-containing DMEM was used as culture medium. One hour after 5 µM TRAM-34 or vehicle (DMSO) was added, cells were irradiated (0 or 2 Gy). After another hour of incubation, a scratch was created by using 20 µL pipette tips (Biosphere® plus, 70.1116.210). Pictures of the scratch were recorded at time points 0, 8 and 24 h after the scratch. The width of scratch was quantified with FIJI (version 2.9.0/1.53t^77^). In general, results of 5 wells per treatment group were averaged for each independent experiment. Migration distance was calculated as follows:

$$Migration\,\,distance= \frac{Distance\,\,of\,\,scratch \left(time \,0\right)-Distance\,\,of\,\,scratch (time \,X)}{2}$$

*TGF-β1 ELISA.* Measuring released TGF-β1 in cell culture supernatants was performed by NS using Mouse TGF-β 1 DuoSet ELISA, DuoSet ELISA Ancillary Reagent Kit 1 and Sample Activation Kit 1 (all R&D Systems, DY1679-05, DY007B, DY010) according to the manufacturer’s instruction. Samples were prepared by plating 187.500 cells/1 mL medium (single-dose irradiation) or 62.500 cells/1 mL medium (fractionated irradiation; lower cell concentrations due to longer incubation periods). Medium used was 0.1% FBS-containing DMEM to reduce the proliferation of cells and avoid cross-contamination of results by bovine TGF-β1 present in FBS. 5 µM TRAM-34 or vehicle (DMSO) was added 4 h after plating of cells. For single-dose irradiation treatment, cells were irradiated (0, 2, 4 or 8 Gy) and supernatants collected 48 h after the irradiation. For fractionated irradiation, cells were irradiated for 5 consecutive days (0, 2 or 4 Gy) and supernatants collected 24 h after the last irradiation. Additionally, cells were detached, counted to correct for residual cell proliferation and used for further RT-PCR measurements.

*RNA isolation and RT-PCR* Sample preparation was described in the previous paragraph. RNA of samples was isolated using the NucleoSpin RNA isolation kit (Macherey–Nagel, #740955.250) according to the manufacturer’s instructions. 20 ng of RNA (as measured by a NanoDrop ND-100 spectrometer) were used for one-step SYBR Green-based reverse transcriptase PCR using the 1 Step RT PCR Green ROX L Kit (highQu) according to the manufacturer’s instruction. Specific fragments (all Quantitect Primer Assay, Qiagen) used, were *KCNN4* (K_Ca_3.1; QT00105672), *TGFBR1* (QT00135828), *MMP2* (QT00116116), *MMP9* (QT00108815) and *CD44* (QT00173404). Results were normalized to the mean abundance of housekeeper genes *GAPDH* (QT01658692) and *PDHB* (QT00163366). Measurements and other steps were performed by NS on a LightCycler480 device (Roche). Crossing point values and melting curves were analyzed using LightCycler 480 software (Roche, version 1.5.0).

*Immunoblotting* Standard protocols were used for SDS polyacrylamide gel electrophoresis (SDS-PAGE) and immunoblotting. In short, cells were lysed with NP-40-based lysis buffer (including protease- and phosphatase-inhibitors), proteins separated by 12% SDS-Page, blotted, and probed against K_Ca_3.1 and against beta-actin as a loading control using a rabbit anti-K_Ca_3.1 antibody (1:1000 in 1% BSA/PBS, Alomone labs, APC-064) and mouse anti-beta-actin antibody (1:20.000 in 1% BSA/PBS, Sigma Aldrich, #A554). Antibody binding was detected with fluorescent anti-rabbit (1:5000 in 1% BSA/PBS, 926–68071, LI-COR Biosciences) or anti-mouse (1:5000 in 1% BSA/PBS, 926–32350, LI-COR Biosciences) secondary antibodies and a LI-COR ODYSSEY FC detection system (LI-COR Biosciences). For blocking specific epitope binding of the anti-K_Ca_3.1 antibody, the antibody was pre- (1 h) and co-incubated with its specific blocking peptide (2 µg blocking peptide per 1 µg of antibody; Alomone labs, BLP-PC064).

*Patch-clamp recording.* Macroscopic on-cell (cell-attached) and whole-cell currents from DMEM medium-grown irradiated (0 or 2 Gy, 90–240 min post-irradiation) SMA-560 cells were recorded by SMH in voltage-clamp mode (10 kHz sampling rate) and 3 kHz low-pass-filtered by an EPC-9 patch-clamp amplifier (Heka, Lambrecht, Germany) using Pulse software (Heka) and an ITC-16 Interface (Instrutech, Port Washington, NY, USA). Borosilicate glass pipettes (~ 5 MΩ pipette resistance; GC150 TF-10, Clark Medical Instruments, Pangbourne, UK) manufactured by a microprocessor-driven DMZ puller (Zeitz, Augsburg, Germany) were used in combination with an STM electrical micromanipulator (Lang, Gießen, Germany). Cells were continuously superfused at 37 °C with NaCl solution (in mM: 125 NaCl, 32 *N*-2-hydroxyethylpiperazine-*N*-2-ethanesulfonic acid (HEPES), 5 KCl, 5 d-glucose, 1 MgCl_2_, 5 CaCl_2_, titrated with NaOH to pH 7.4) additionally containing 0 or 200 µM of the K_Ca_3.1 K^+^ channel activator 1-EBIO and 0 or 1 µM of TRAM-34. The pipette solution contained in on cell-mode (in mM) 130 KCl, 32 HEPES, 5 d-glucose, 1 MgCl_2_, 5 CaCl_2_, titrated with KOH to pH 7.4. and in whole-cell mode (in mM) 140 K-d-gluconate, 5 HEPES, 5 MgCl_2_, 1 K_2_-EGTA, 1 K_2_-ATP, titrated with KOH to pH 7.2.

Currents were elicited by 41 voltage square pulses (700 ms each) from 0 mV holding potential to voltages between − 100 mV and + 100 mV (on-cell) or + 75 mV (whole-cell) delivered in 5 mV increments. Clamp voltages refer to the cytosolic face of the plasma membrane and were not corrected (on-cell) or corrected by adding − 10 mV (whole cell) for the liquid junction potential between pipette and bath solution. For analysis, macroscopic on-cell currents were averaged between 100 and 700 ms of each voltage sweep. Inward currents are defined as influx of cations into the cells (or efflux of anions out of the cell), depicted as downward deflections of the current tracings, and defined as negative currents in the current voltage relationships. Macroscopic on-cell conductance was calculated for the inward currents between − 100 mV and + 25 mV clamp voltage. The 1-EBIO-stimulated and TRAM-34-sensitive increase in macroscopic on-cell conductance and the associated change in current reversal potential were used as a measure of functional K_Ca_3.1 channel expression in the plasma membrane. For single channel analysis, currents were continuously on-cell recorded (10 kHz sampling rate, 3 kHz low-pass-filtering) for several seconds at clamp voltages between -100 mV and + 100 mV (10 mV increments) and unitary current transitions were analyzed as a measure of channel amplitude. In addition, single channel activity was assessed during wash-in and wash-out of TRAM-34 (1 µM).

*Animal model.* All animal experiments were performed in accordance to the laboratory animal research guidelines, authorized by the local ethics committee for Animal Research (Regierungspräsidium Tübingen, Germany) with the registration code R13/18G and performed by NS or KG. This manuscript complies with the ARRIVE guidelines 2.0^78^. VM/Dk mice were kept under standard SPF conditions on a dark–light cycle of 12 h with humidity of 55 ± 10%, temperatures of 22 ± 2 °C, and ad libitum food and water supply.

*Tumor transplantation* Two-to-six-month-old male and female mice (60% male; mean body weight: 26.6 g, standard deviation: 4.5 g) were anaesthetized using fentanyl (0.05 mg/kg b.w. [body weight]), midazolam (5 mg/kg b.w.) and medetomidine (0.5 mg/kg b.w.) intraperitoneally. Body temperature was stabilized via external heating mats. Mice were placed in a stereotactic fixation device (Stoelting) and a burr hole was drilled 2 mm lateral of the bregma to ensure transplantation into the right striatum as described previously^36^. After ensuring no excess bleeding from the burr hole, the needle of a Hamilton syringe was inserted to a depth of 3 mm from the level of the skull surface, after which it was pulled out 0.5 mm to ensure a final depth of injection of 2.5 mm. 5 × 10^3^ SMA-560 cells in phosphate-buffered saline were injected slowly over 1 min. After the injection and an additional waiting period of 2 min, the syringe was slowly withdrawn. Tissue adhesive (Histoacryl®, B. Braun) was used for closing the burr hole and the skin sutured. After the procedure, antidotes naloxone (1.2 mg/kg b.w.), flumazenil (0.5 mg/kg b.w.) and atipamezole (0.5 mg/kg b.w.) were administered subcutaneously to end anesthesia. Carprofen analgesic treatment (0.5 mg/kg b.w.) was administered subcutaneously right before the surgical intervention started and for three additional days after it. All drugs (except for fentanyl, which was purchased separately: Fentadon 50 µg/mL, Eurovet Animal Health B.V.) were provided by the facility of animal welfare of the University of Tübingen.

Afterwards, the animals were observed and weighed daily for the first 7 days after the injection or if any sign of distress was present. Otherwise, monitoring frequency was twice weekly. Criteria of experiment termination were defined as reaching a cumulative animal observation score of 5 or higher (see Supplementary Table S1 for short descriptions of all components of the score). Long-term surviving animals from the overall survival experiments were taken off the experiment after a maximum of 150 days. Animals were sacrificed using CO_2_ asphyxia in their cages. Afterwards, the brains of the animals were extracted and fixated in phosphate-buffered 4.5% formalin solution. All steps were performed by NS or KG.

For power calculations in the orthotopic SMA-560 glioma mouse model, we estimated approximately 5% of animals surviving more than 7 weeks, as has been reported previously^36^. Required mouse numbers per treatment group were estimated for alpha and beta errors of 5% and 20%, respectively, and a treatment-associated increase in ≥ 7-weeks-survivors from 5 to 20%. Such quadruplication of “long-term” survivors was considered scientifically relevant when extrapolating to the clinical situation of glioblastoma patients. The sample size calculation led us to aim for 12 animals per treatment group (excluding 20% reserve animals for the substitution of animals with failed tumor cell engraftment). In the end, we slightly fell short of 12 animals per treatment group due to changes at the beginning of the study. In detail, we initially planned to deliver 10 fractions of 2 Gy irradiation, which was not feasible as most animals reached the stopping criteria before the end of treatment. To compensate, we initially lowered the number of injected tumor cells (5 × 10^2^) which led to inconsistent tumor engraftment. Hence, we changed back to injecting 5 × 10^3^ cells and treating animals with 5 × 4 Gy of irradiation. Moreover, we excluded one batch of 8 animals from further analyses as none of the animals showed any sign of tumor development, hinting at technical issues during the surgery. One animal (TRAM-34 group) was excluded as bleeding from the burr hole at the time of tumor cell injection potentially interfered with tumor engraftment. Exclusion criteria were not stated a priori.

*Treatment of animals* Treatment of the animals started on day 7 after tumor cell injection and was performed by NS or KG. Experimental mouse batches, i.e., animals transplanted in one session, were randomized (https://www.randomizer.org/) to one of the following treatments, that were administered daily for five consecutive days (days 7–11): control (5 × 0 Gy and 5 × miglyol intraperitoneally), irradiation (IR) only (5 × 4 Gy and 5 × miglyol i.p.), TRAM-34 only (5 × 0 Gy and 5 × 120 mg/kg b.w. TRAM-34 in miglyol i.p.) or combined IR + TRAM-34 treatment (5 × 4 Gy and 5 × 120 mg/kg b.w. TRAM-34 in miglyol i.p.). TRAM-34 or vehicle (miglyol) was injected 6 h before irradiation of the animals. As mentioned previously, experiments were blinded in regard to drug treatment. Irradiation was conducted as previously described^14^. In short, all animals were anaesthetized with isoflurane (Isofluran CP®, CP-Pharma). Animals from the irradiation only or IR + TRAM-34 groups were placed under the linear accelerator and body parts, except for parts of the right hemisphere, were protected by a lead shield (Fig. 1a, right; for target volume and dose distribution, see Edalat et al.^14^). Irradiation was performed with a 6 MV linear accelerator (LINAC SL25 Philips) at room temperature and a dose rate of 3.5 Gy/min.

*Immunohistochemistry.* All brains of an experimental batch (see above) were extracted on the same day by NS. Time of extraction was planned as of 14 days after the last day of treatment or as soon as the first animal within a batch reached the termination criteria (range: 3–14 days post-treatment). 4 animals (1 per group) were only injected with 5 × 10^2^ SMA-560 cells due to an earlier protocol (all animals developed a visible tumor). H&E and immunohistochemical stainings were performed by IGM and LQM. For histology, 3–5 µm-thick sections were cut and stained with haematoxylin and eosin (H&E). Immunohistochemistry was performed on brains of animals displaying an H&E-Score ≥ 1 on an automated immunostainer (Ventana Medical Systems, Inc.) according to the company’s protocols for open procedures with slight modifications. The slides were stained with the antibodies Iba1 (Abcam, Cambridge, UK), CD68 (ab125212, Abcam, Cambridge, UK) CD3 (clone SP7, DCS Innovative Diagnostik-Systeme GmbH u. Co. KG, Hamburg, Germany), CD8 (clone C8/144B; Dako, Glostrup, Denmark) and FoxP3 (236A/E7, Abcam, Cambridge, UK). Appropriate positive and negative controls were used to confirm the adequacy of the staining. All samples were scanned with the Ventana DP200 (Roche, Basel, Switzerland) and processed with the Image Viewer MFC Application. Final image preparation was performed with Adobe Photoshop CS6. The tumor histology and the tumor microenvironment were scored as follows:

H&E: Score 0: no tumor or histological alterations were detected; Score 1: gliosis, no tumor or necrosis detected; Score 2: small tumors without clear tumor borders accompanied by gliosis, but no necrosis detected; Score 3: medium to large tumors with clear tumor borders accompanied by gliosis and necrosis in the tumor core. Additional occurrence of multiple satellite tumors (foci of tumor not connected to the main body tumor, related to invasiveness) were scored separately.

Iba1: Score 0: no alterations, the microglia showed few and thin ramifications; Score 1: mild increase of the reactive microglia (shorter and thicker ramifications) and; Score 2: moderate to prominent reactive microglia accompanied by phagocytic microglia (loss of the ramification and globular inflated cells body) in the tumor core. Some animals presented with macrophage pseudopalisading around the tumor.

CD68: Score 0: no alterations were detected, the microglia present showed few and thin ramifications; Score 1: mild focal increase of the CD68 + macrophages; Score 2: moderate to prominent increase of CD68 + macrophages. Pseudopalisading of the macrophages around the tumor is present in some tumors.

CD3: Score 0: no presence of CD3 positive cells; Score 1: mild to moderate presence of CD3 positive cells; Score 2: prominent presence of CD3 positive cells.

CD8 and FoxP3: Score 0: no presence of positive cells; Score 1: mild presence of positive cells; Score 2: moderate presence of positive cells.

*Blood counts* Blood samples were collected from tail vein or retro-orbital sinus in isoflurane anesthesia (for the latter one) by NS. Time points of sample collection were: first, for a baseline measurement on the day before treatment initiation (day 6 post-surgery); second, after the last round of irradiation treatment (day 11 post-surgery); and lastly, 3 weeks after the last day of treatment or if the stopping criteria for individual animals were reached (day 15–32 post-surgery). Blood samples were analyzed with a Hematology Analyzer HM5 (Abaxis) right after sample collection.

*Data visualization and statistical analysis* Data visualization and statistical tests were performed using GraphPad Prism (version 9.4.1) by NS and SMH. Statistical tests were performed using (mean) values of independent experiments (in vitro) or animals (in vivo experiments). Specifics are given in the respective figure legend.

### Supplementary Information

Supplementary Information.

## Data Availability

Data is available upon reasonable request from the corresponding author.
