# Supplementary material for: Efficacy of combined tumor irradiation and KCa3.1-targeting with TRAM-34 in a syngeneic glioma mouse model
Source: Sci Rep. 2023 Nov 23;13:20604. doi: 10.1038/s41598-023-47552-4 (PMC10667541; doi:10.1038/s41598-023-47552-4)
Supplement: Supplementary file 1 — Supplementary Information. [file 41598_2023_47552_MOESM1_ESM.pdf]

Efficacy of Combined Tumor Irradiation and $K_{Ca}3.1$-Targeting with TRAM-34 in a Syngeneic Glioma Mouse Model

Nicolai Stransky[1,2], Katrin Ganser[1], Leticia Quintanilla-Martinez[3, 4], Irene Gonzalez-Menendez[3, 4], Ulrike Naumann[5,6], Franziska Eckert[1,7], Pierre Koch[8], Stephan M. Huber[1, *] and Peter Ruth[2]

Supplementary Information

Supplementary Table 1: Components of the scoring system used to assess stopping criteria

Score	Weight loss	Animal behavior	Physical appearance	Grimace Scale	Local reactions	Neurologic signs
1	> 0 %	Less attentive	Fur care impaired	1-2	Redness, edema	-
2	-	Lethargy	Incrustations	-	Automutilation	Tremor
3	> 10 %	Apathy	Exsiccosis, cloudy eyes	> 2	Skin lesions (e.g., bleeding)	-
4	-	-	-	-	Infection (pus)	-
5	> 20 %	Hunched back	-	> 2 for 72 hours despite analgesia	Tumor ulceration	Seizures, hemiplegia, paresis

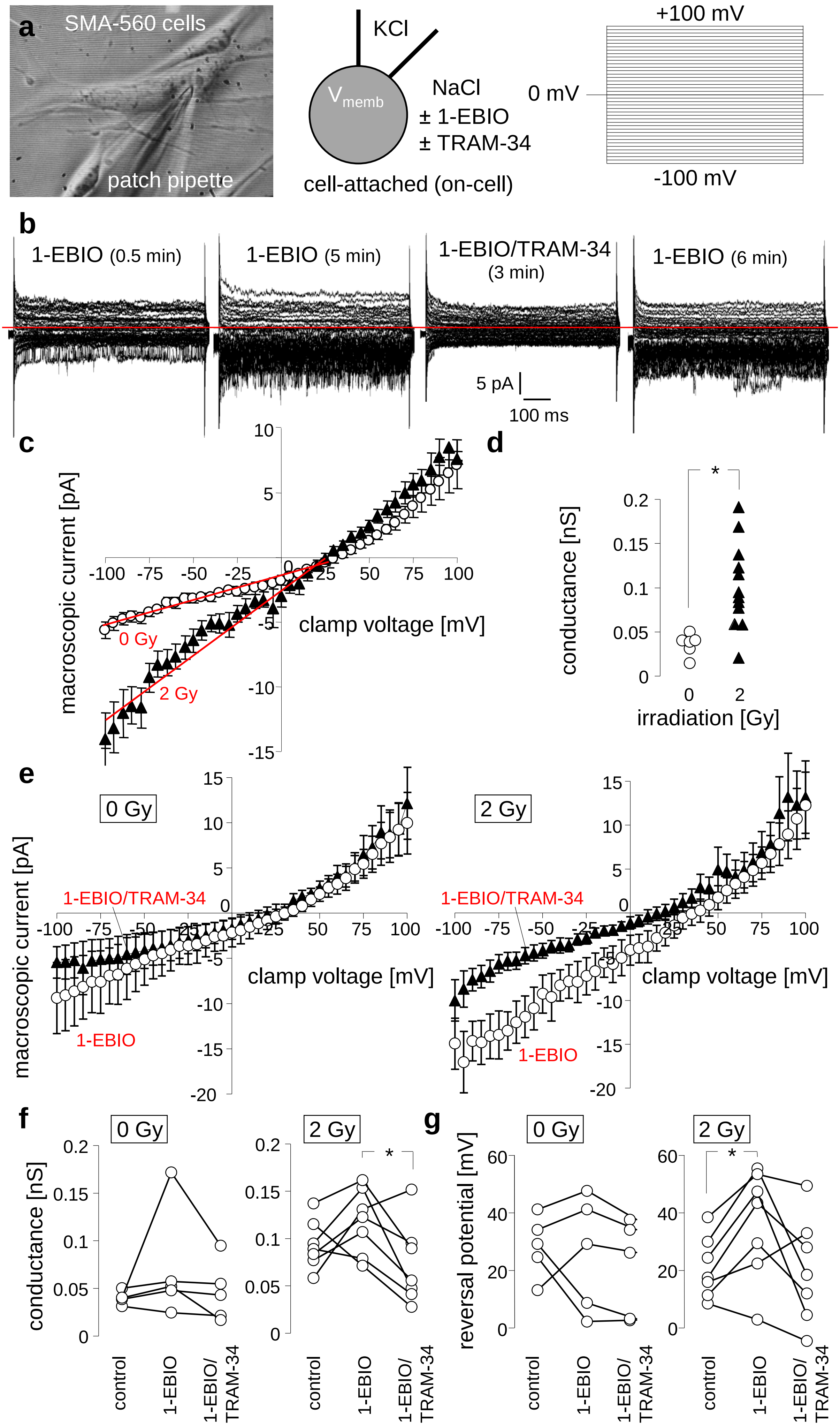
a
SMA-560 cells
patch pipette
KCl
V memb
NaCl
± 1-EBIO
± TRAM-34
cell-attached (on-cell)
+100 mV
0 mV
-100 mV
b
1-EBIO (0.5 min)
1-EBIO (5 min)
1-EBIO/TRAM-34 (3 min)
1-EBIO (6 min)
5 pA
100 ms
c
macroscopic current [pA]
clamp voltage [mV]
0 Gy
2 Gy
d
conductance [nS]
irradiation [Gy]
e
0 Gy
2 Gy
1-EBIO/TRAM-34
1-EBIO
macroscopic current [pA]
clamp voltage [mV]
f
0 Gy
2 Gy
conductance [nS]
control
1-EBIO
1-EBIO/ TRAM-34
g
reversal potential [mV]
0 Gy
2 Gy

Supplementary Figure S1: Irradiation induces upregulation of $K_{Ca}3.1$ K^+ currents in the plasma membrane of SMA-560 cells. **a**, Micrograph of SMA-560 cells during recording (left), and schemes of applied ion compositions, $K_{Ca}3.1$ channel modulators (middle), and voltage pulse protocol (right) used to record $K_{Ca}3.1$-generated macroscopic on-cell (cell-attached) currents from irradiated (0 or 2 Gy) SMA-560 cells cultured in DMEM medium. **b**, On-cell current tracings recorded as in **a** in a 2 Gy-irradiated SMA-560 cell direct after wash-in of the $K_{Ca}3.1$ K+ channel opener 1-EBIO (200 µM), after long term-stimulation with 1-EBIO (middle left), during additional superfusion of TRAM-34 (1 µM, middle right) and after wash-out of TRAM-34 (right). Red lines indicate zero current level. **c**, Current-voltage relationships and, **d**, derived conductance of 0 Gy (open circles, n = 6)- and 2 Gy (closed triangles, n = 12)-irradiated SMA-560 cells recorded under control conditions. Data are means ± SE in **c** and individual values in **d**. **e**, Dependence of mean (± SE) current on voltage in 0 Gy (n = 5, left)- and 2 Gy (n = 7, 167 ± 15 min post irradiation, right) -irradiated SMA-560 cells recorded as in **a** during bath application of 1-EBIO (open circles) and co-application of 1-EBIO and TRAM-34 (closed triangles). **f**, Paired conductances and, **g**, paired current reversal potentials recorded in 0 Gy (left)- and 2 Gy (right)-irradiated SMA-560 cells under control conditions, during application of 1-EBIO, and during superfusion with 1-EBIO and TRAM-34. Conductances in **d** and **f** were calculated by linear regression for the inward current range depicted in **c** by red line. * indicates $_zP \leq 0.05$ as calculated by Welch-corrected (**d**) and paired (**f**, **g**) two-tailed t-test with Bonferroni correction for z = 1 (**d**) and z = 2 (**f**, **g**) pairwise comparisons.

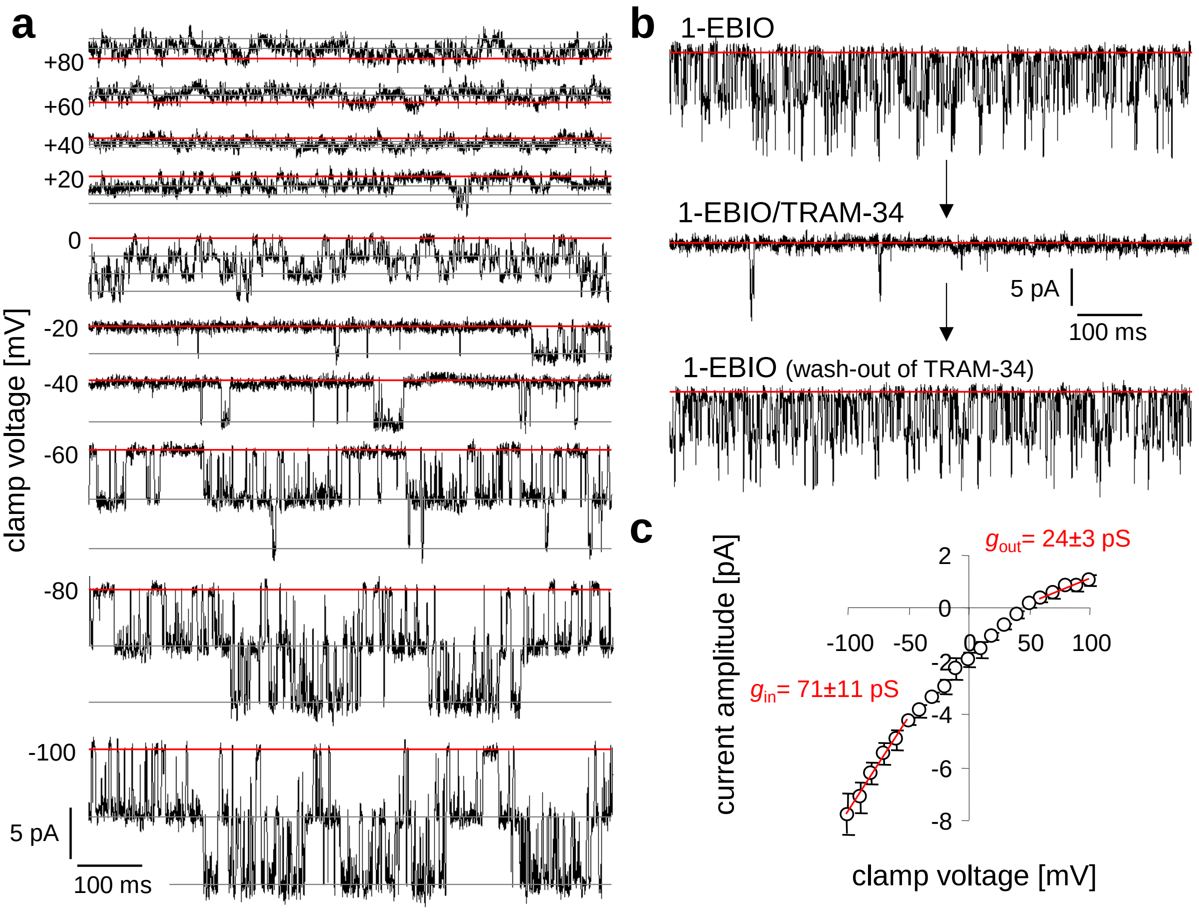

Supplementary Figure S2: The TRAM-34 sensitive current fraction is generated by an intermediate conductance, voltage-independent ion channel type with inwardly rectifying amplitude-voltage relationship. **a**, Current tracings from a 2 Gy-irradiated SMA-560 cell recorded as in Supplementary Figure S1A at various clamp voltages (as indicated). **b**, Current

tracings obtained as in (**a**) before (top), during (middle), and after (wash-out) bath application of TRAM-34 (1 µM) from a 2 Gy-irradiated and 1-EBIO (200 µM)-stimulated SMA-560 cell. **c**, Relationship between mean (± SE, n = 3) channel amplitude and holding potential. Red and grey lines in (**a**, **b**) indicate zero current and distinct current levels of open channel(s). Red numbers in **c** give the single channel conductance at negative (-100 to -50 mV) and positive (+50 to +100 mV) clamp voltage, respectively.

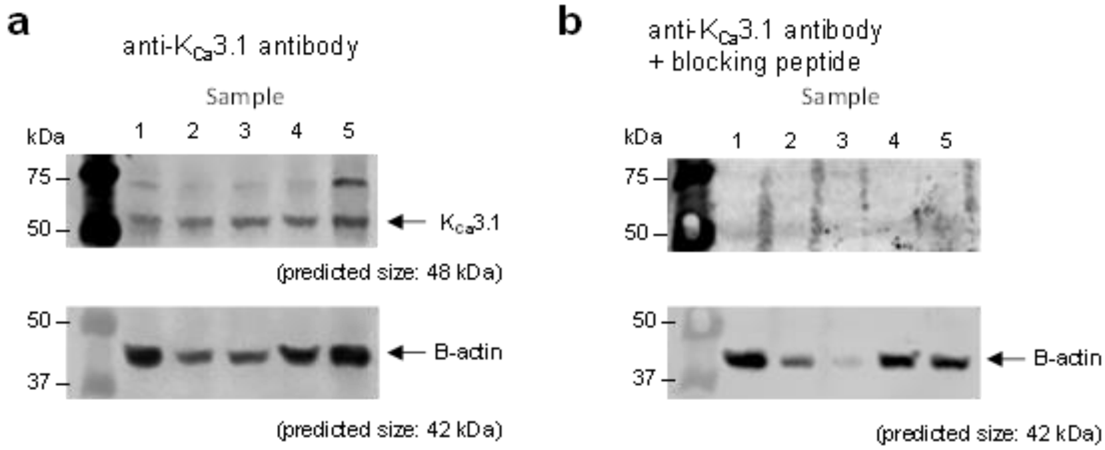

Supplementary Figure S3: Specific K_{Ca}3.1 protein staining in SMA-560 cells. **a**, Staining of five different samples of SMA-560 cells, grown in DMEM medium, with anti-K_{Ca}3.1 antibody (shown above) and b-actin as a loading control (shown below) shows staining of K_{Ca}3.1 at the predicted size. **b**, No specific staining of samples after pre-incubation of antibody with its blocking peptide, indicating specific staining in **a**. Note that samples 2 and 3 showed weaker staining of b-actin (especially in **b**), and hence are only interpretable with caution.

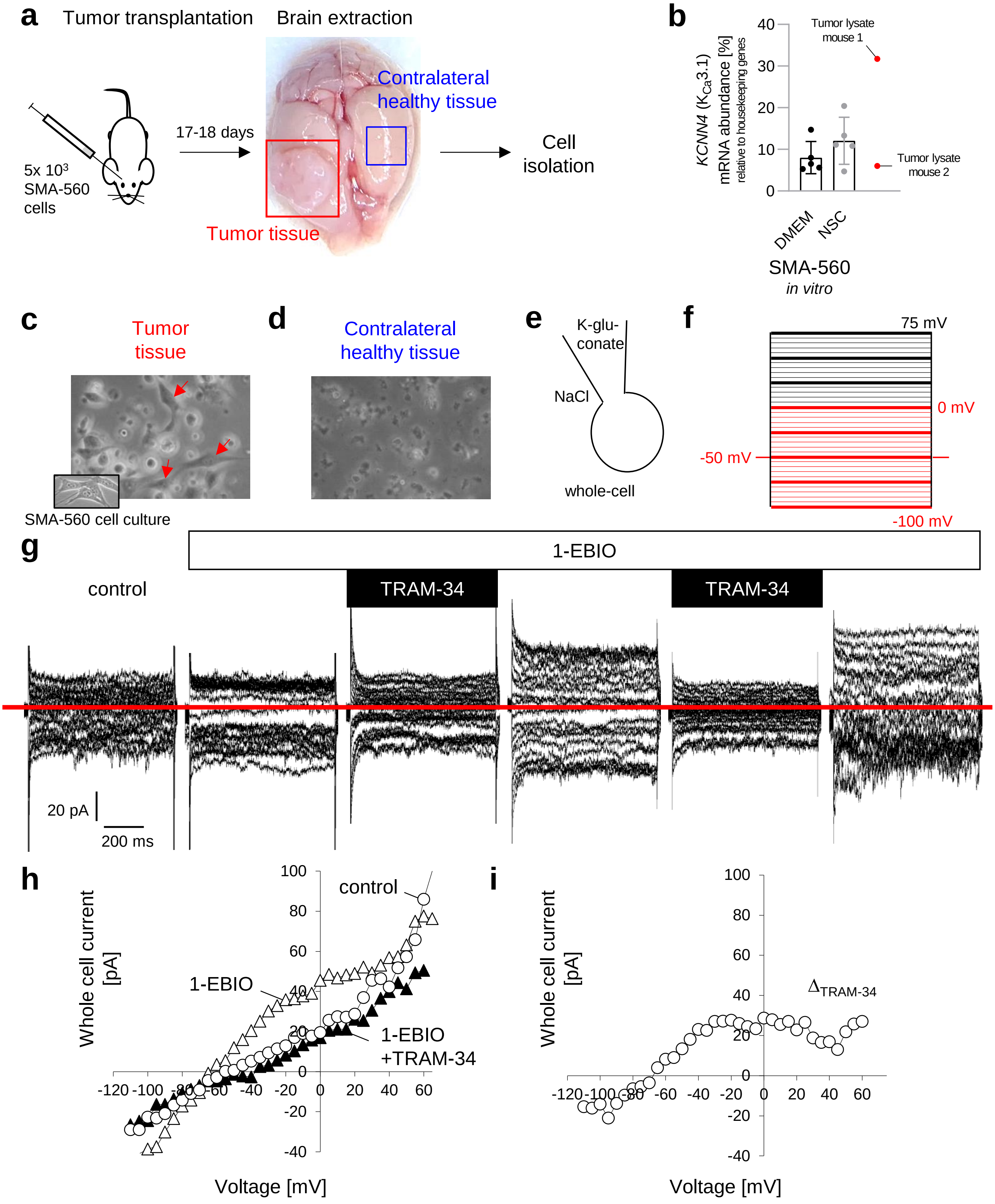

Supplementary Figure S4: *Ex vivo* SMA-560 cells functionally express $K_{Ca}3.1$ after tumor formation in VM/Dk mice. **a**, Schematic of tumor transplantation and subsequent SMA-560 cell isolation from tumor tissue (indicated by red rectangle) and the contralateral healthy brain tissue (indicated by blue rectangle). **b**, mRNA abundances of *KCNN4* in tumor lysates are similar to those observed in SMA-560 cell cultures *in vitro*. **c**, After isolating cells (as described in Ganser, K., *et al*., IJC (2022)) and plating them for 1-2 hours in DMEM medium, several cells

from the tumor tissue adhered to the culture plates (indicated by red arrows). Cell phenotype of adhering cells closely matches phenotype of SMA-560 cells cultured *in vitro* (as shown in the black box). **d**, No cells from the healthy tissue adhered to the culture plates, even after several hours. **e**, **f**, Drawings depicting ionic compositions of pipette and bath solution (**e**), and voltage-clamp pulse protocol (**f**) used for the recording of whole-cell currents from *ex vivo* SMA-560 cells isolated from orthotopic tumors. **g**, Whole-cell current tracings recorded before (outer left) and during bath application of the $K_{Ca}3.1$ opener 1-EBIO alone (500 µM, 2nd, 4th, 6th tracings) and during co-application of the $K_{Ca}3.1$ inhibitor TRAM-34 (200 nM, 3rd and 5th tracings). For clarity, only current sweeps elicited by voltage steps between -100 mV and 0 mV (red voltage range in (**f**)) are given. **h**, Current/voltage-relationships showing the whole-cell currents of an *ex vivo* SMA-560 cell recorded as in (**e-g**) before (open circles), during subsequent wash-in of 1-EBIO (open triangles) and TRAM-34 (closed triangle). **i**, Current/voltage-relationship of the TRAM-34-sensitive whole-cell current fraction (data from (**h**)), Note the current reversal potential close to K^+ equilibrium potential (EK) and the inwardly rectifying current/voltage-relationship typical for $K_{Ca}3.1$.

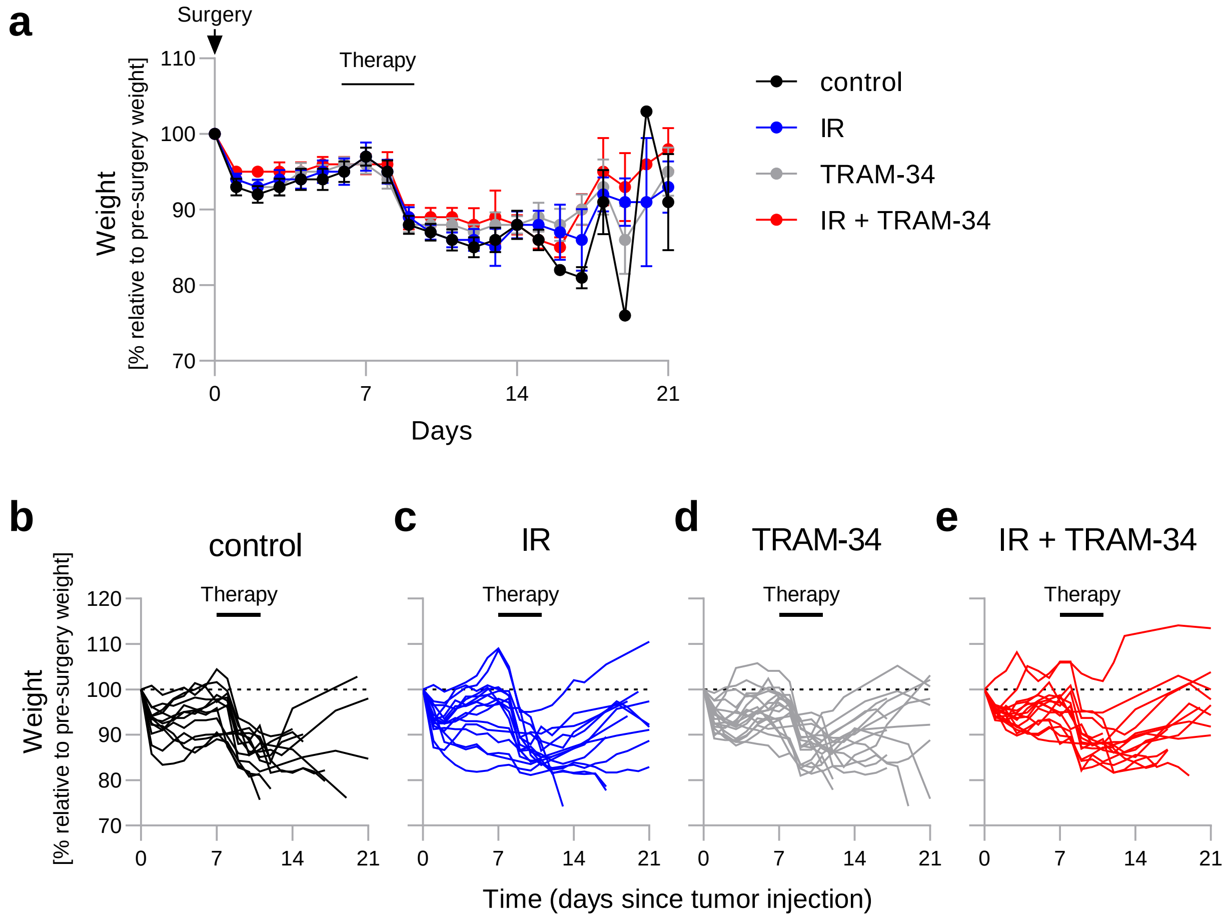

Supplementary Figure S5: Weight change of animals. **a**, Depicted are mean values ± s.e.m. of all animals per treatment group relative to their respective weight before surgery. Some variability in the latter part of the curve may arise due to few mice having weight records on that specific day post-surgery. **b**-**e**, Weight change of individual animals per treatment group. Dashed line represents weight before surgery.

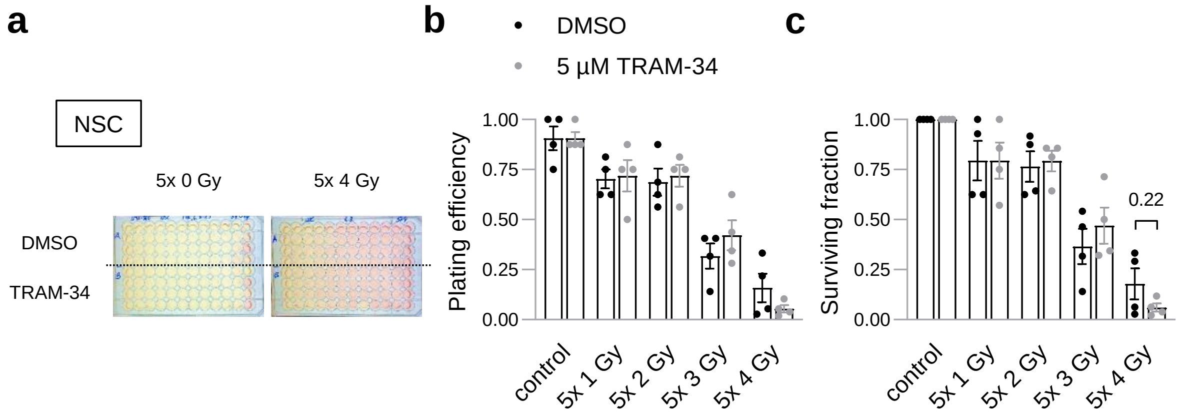

Supplementary Figure S6: TRAM-34 does not affect clonogenic survival after fractionated irradiation in SMA-560 cells *in vitro*. Results shown are for SMA-560 cells grown in stem-like cell-enriching NSC medium. **a**, Representative image of limited dilution assay on day 18. **b**, Plating efficiency and, **c**, surviving fraction for cells after fractionated irradiation (5x 0, 1, 2, 3 or 4 Gy) and TRAM-34 treatment (0 or 5 µM). Individual values of 4 independent experiments and mean values ± s.e.m. are depicted. Number (**c**) indicates *P* values as calculated by Welch-corrected two-tailed t tests.

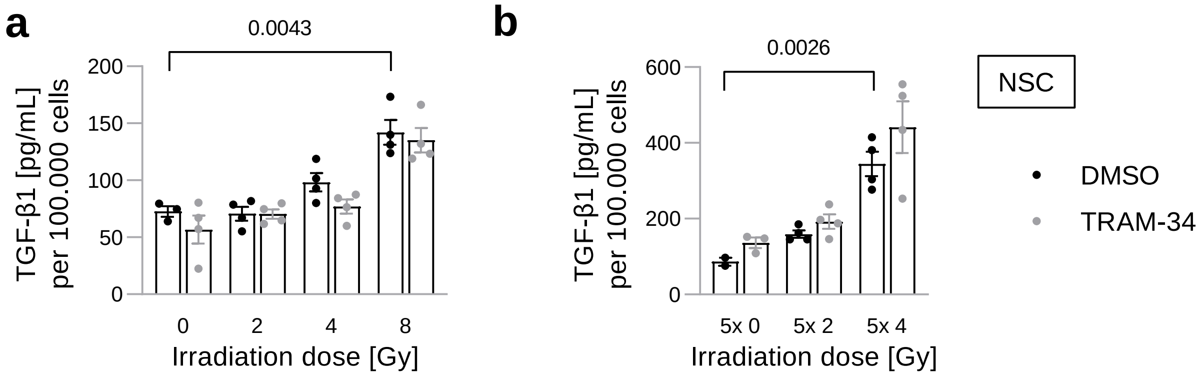

Supplementary Figure S7: Explorative analysis of TGF-β1 secretion per 100.000 cells after single-dose (**a**) or fractionated (**b**) irradiation in stem-like cell-enriched SMA-560 cells, which both increase TGF-β1 secretion significantly. Additional TRAM-34 exposure has no effect on TGF-β1 secretion. Depicted are individual values from 2-4 independent experiments and mean values ± s.e.m. Numbers (**a**, **b**) indicate *P* values as calculated by Welch-corrected two-tailed t tests.

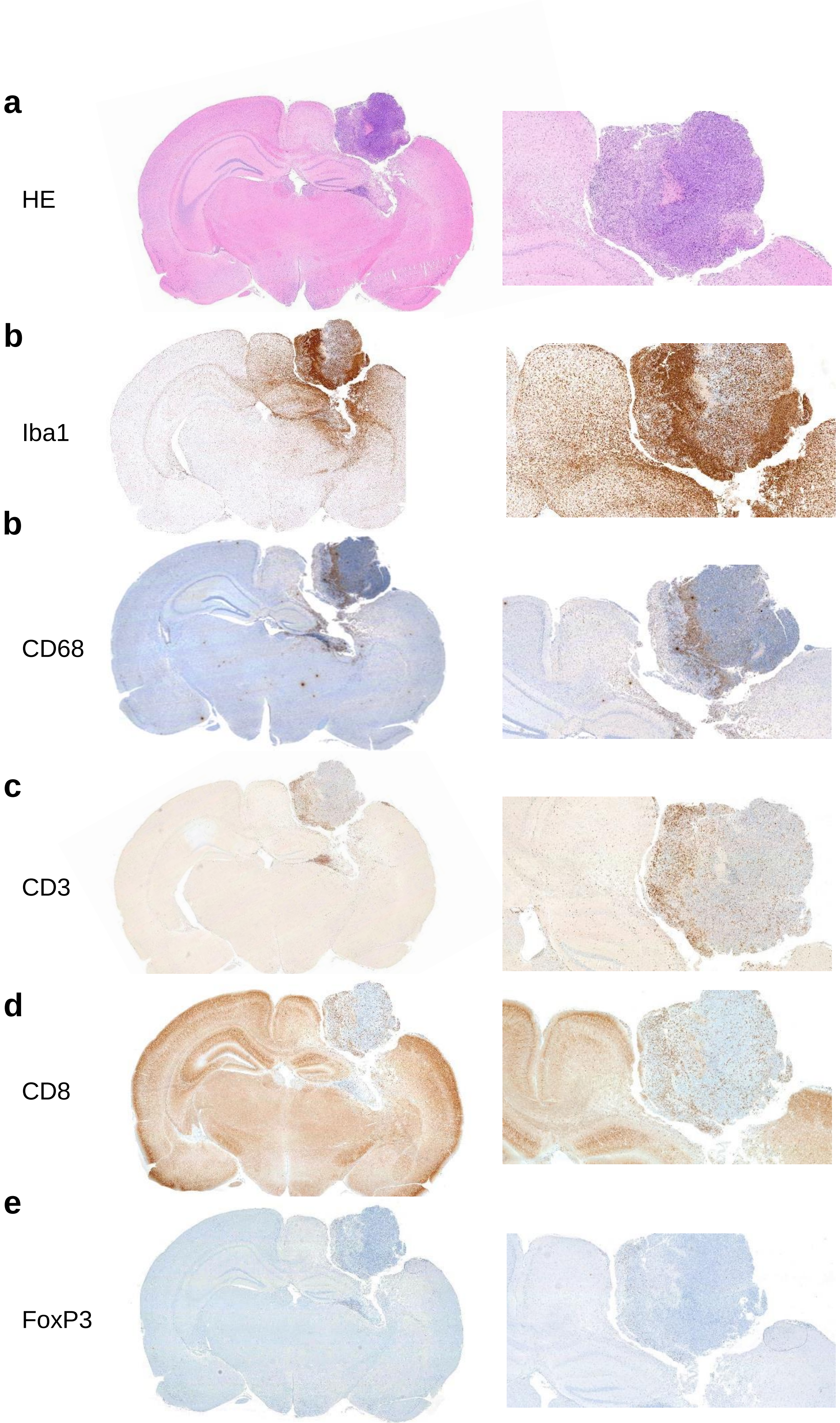
a
HE
b
Iba1
b
CD68
c
CD3
d
CD8
e
FoxP3

Supplementary Figure S8: Distribution of Iba1^{+} and CD68^{+} microglia/macrophages or CD3+, CD8^{+} and FoxP3^{+} T cells (each micrograph depicts a representative image of an animal with a respective staining score of 2). The CD3^{+}, CD8^{+} and FoxP3^{+} T cells were more abundant in the tumor periphery than in the tumor center.

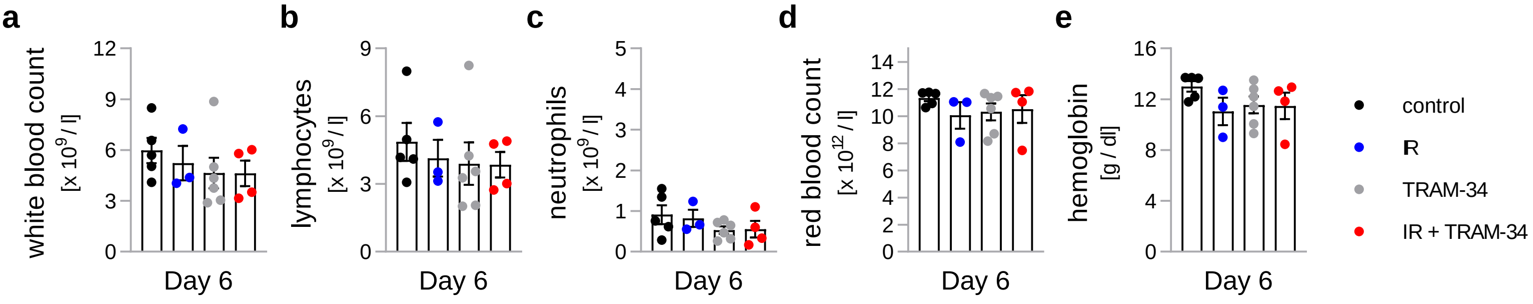

Supplementary Figure S9: Blood counts before first day of treatment. Mean values are evenly distributed among each treatment group. Depicted are individual values from 3-6 animals and means ± s.e.m. Statistical test used to test for differences before randomization was ordinary one-way ANOVA and Tukey’s multiple comparisons test.

Sample Name: Tram34

===

Acq. Operator : Seq. Line : 97
Acq. Instrument : Instrument 1 Location : Vial 95
Injection Date : 3/5/2020 10:39:10 AM Inj : 1
Inj Volume : 5.0 µl
Acq. Method : C:\CHEM32\1\DATA\DEF_LC 2020-03-03 18-50-02\C18STD.M

Additional Info : Peak(s) manually integrated

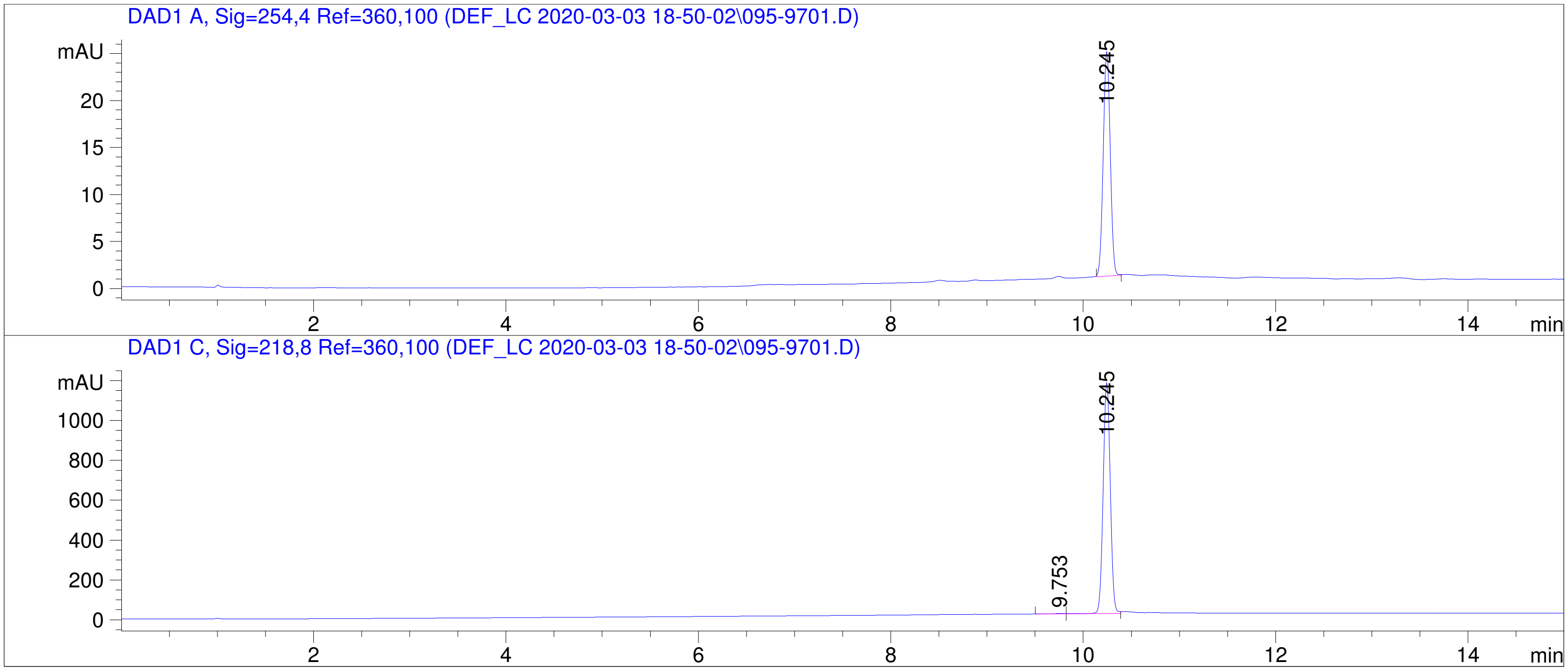

===

Area Percent Report

===

Sorted By : Signal
Multiplier: : 1.0000
Dilution: : 1.0000
Use Multiplier & Dilution Factor with ISTDs

Signal 1: DAD1 A, Sig=254,4 Ref=360,100

Peak #	RetTime [min]	Type	Width [min]	Area [mAU*s]	Height [mAU]	Area %
1	10.245	BB	0.0793	121.61677	23.91916	100.0000

Totals : 121.61677 23.91916

Signal 2: DAD1 C, Sig=218,8 Ref=360,100

Peak #	RetTime [min]	Type	Width [min]	Area [mAU*s]	Height [mAU]	Area %
1	9.753	BV	0.1473	15.18765	1.41465	0.2533
2	10.245	VV	0.0821	5979.92627	1160.63660	99.7467

Totals : 5995.11392 1162.05125

===

*** End of Report ***

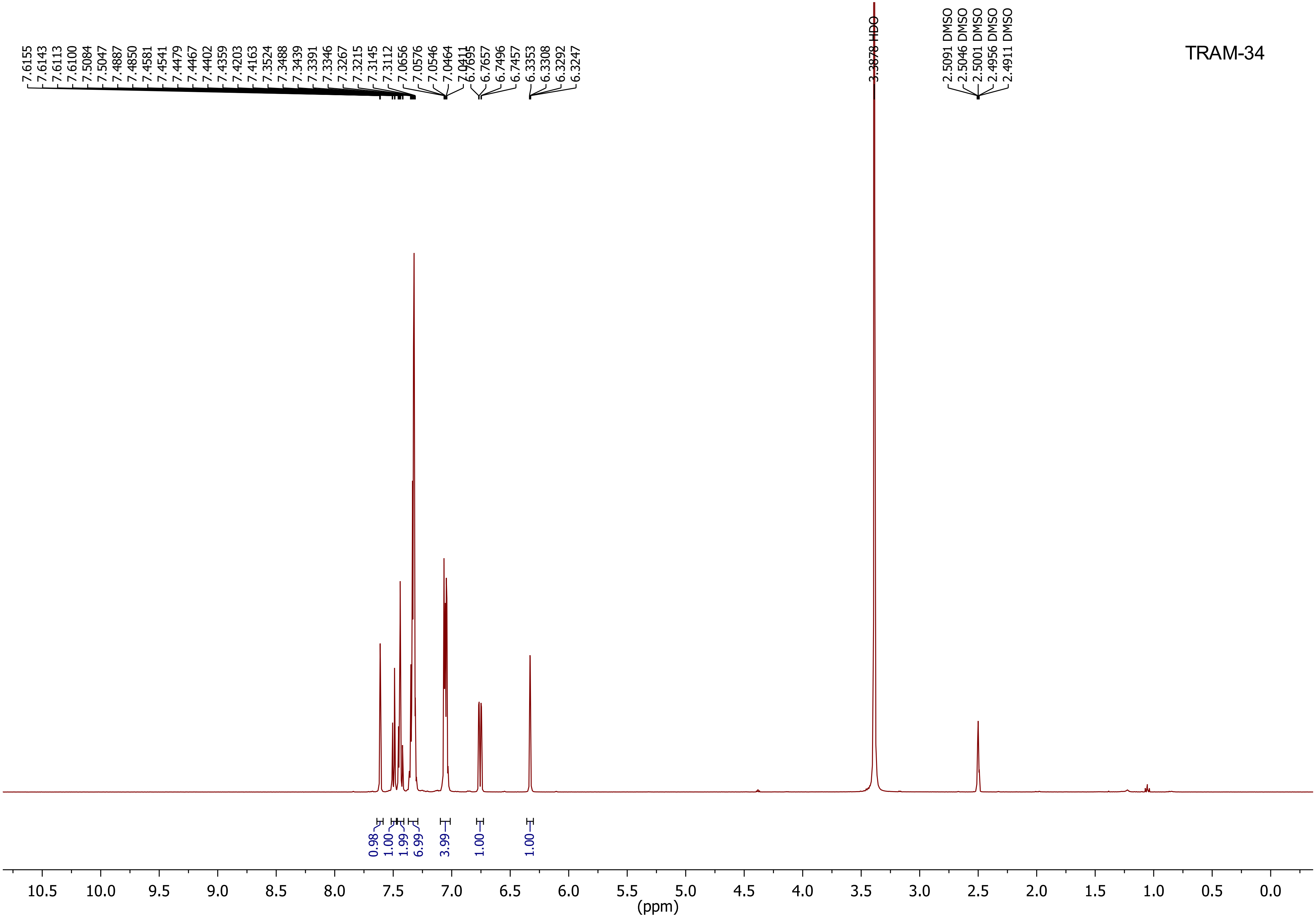
TRAM-34
7.6155
7.6143
7.6113
7.6100
7.5084
7.5047
7.4887
7.4850
7.4581
7.4541
7.4479
7.4467
7.4402
7.4359
7.4203
7.4163
7.3524
7.3488
7.3439
7.3391
7.3346
7.3267
7.3215
7.3145
7.3112
7.0656
7.0576
7.0546
7.0464
7.0411
6.7695
6.7657
6.7496
6.7457
6.3353
6.3308
6.3292
6.3247
3.3878 HDO
2.5091 DMSO
2.5046 DMSO
2.5001 DMSO
2.4956 DMSO
2.4911 DMSO
0.98
1.00
1.99
6.99
3.99
1.00
1.00
10.5
10.0
9.5
9.0
8.5
8.0
7.5
7.0
6.5
6.0
5.5
5.0
4.5
4.0
3.5
3.0
2.5
2.0
1.5
1.0
0.5
0.0
(ppm)

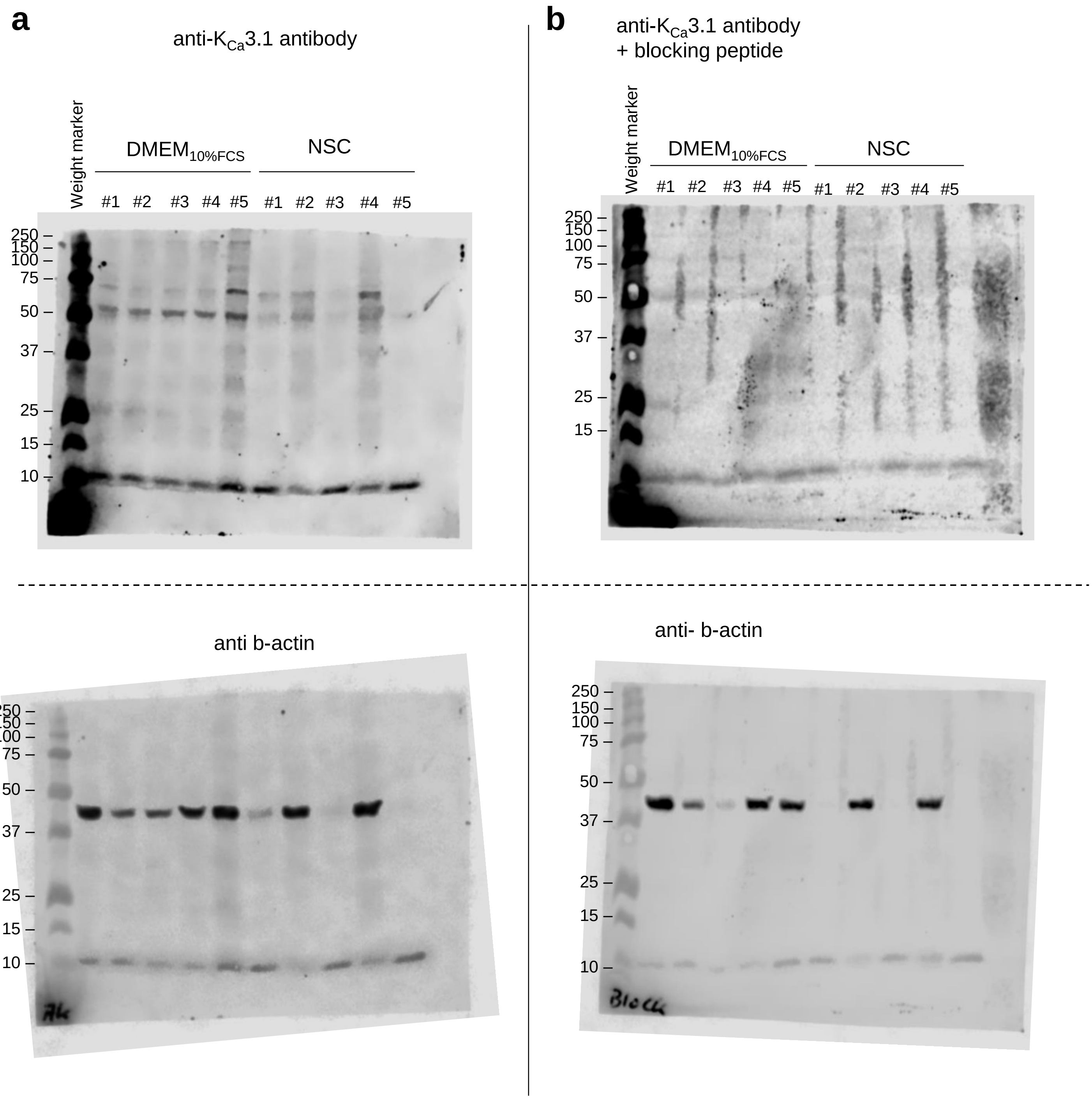
a
anti-K$_{Ca}$3.1 antibody
Weight marker
DMEM$_{10\%FCS}$
NSC
#1 #2 #3 #4 #5 #1 #2 #3 #4 #5
250
150
100
75
50
37
25
15
10
b
anti-K$_{Ca}$3.1 antibody
+ blocking peptide
Weight marker
DMEM$_{10\%FCS}$
NSC
#1 #2 #3 #4 #5 #1 #2 #3 #4 #5
250
150
100
75
50
37
25
15
anti b-actin
250
150
100
75
50
37
25
15
10
anti- b-actin
250
150
100
75
50
37
25
15
10
